# Supplementary material for: Lyn kinase regulates egress of flaviviruses in autophagosome-derived organelles
Source: Nat Commun. 2020 Oct 15;11:5189. doi: 10.1038/s41467-020-19028-w (PMC7564011; doi:10.1038/s41467-020-19028-w)
Supplement: Supplementary file 1 — Supplementary Information [file 41467_2020_19028_MOESM1_ESM.pdf]

Supplementary information

Supplementary note 1

Expression of acylated Lck is able to rescue Lyn-deficiency during Dengue infection

Within the SFK members, Lyn, Lck and Hck comprise the SrcB subfamily, displaying significant overlap in homology, fatty acylation and subcellular distribution. To determine whether a related member within this subfamily can rescue Lyn-deficiency, we engineered Vero cells to stably express inducible Lck in the $Lyn^{-/-}$ background. Cells expressing the wild-type, palmitoylation-deficient mutant (C3S) and an N-terminal truncation (ΔSH4) that cannot be myristoylated or palmitoylated were generated by doxycycline-inducible lentiviral transduction. All the Lck constructs could be expressed in the $Lyn^{-/-}$ cells in an inducible manner as verified by immunoblotting (**Figure S8a**). We challenged these cells with Dengue and Zika virus and measured viral titers. Wild-type Lck but not the acylation-deficient variants (C3S or ΔSH4) could rescue viral titers in the $Lyn^{-/-}$ cells, as quantitated by plaque assays. However, only a partial rescue of virus production could be achieved with wt Lck in Zika-infected cells, suggesting that specific interactions between viral proteins and the SFKs might be necessary in transport of progeny virions (**Figure S8b**). In parallel, secretion alone was measured in cells expressing the corresponding VLPs. Again, wild-type Lck but not the acylation-deficient variants (C3S or ΔSH4) could rescue the secretion defect in $Lyn^{-/-}$ cells quantitated by the appearance of E-protein in supernatants (**Figure S8c**). To confirm the effect of Lck on the subcellular distribution of virus particles, we performed biochemical fractionations of secretory organelles from homogenates of Dengue-infected cells (**Figure S8d**). Reminiscent of Lyn activity, wild-type Lck and viral E-protein co-migrated with markers of recycling endosomes and plasma membrane. On the other hand, in the presence of the C3S or ΔSH4 mutant, Lck and viral E-protein

co-sedimented with lysosomal fractions. These data indicate that SFK members with similar biophysical properties and membrane affinity can facilitate virus secretion.

Materials and Methods:

MTT assay to determine cell viability

2×10^4 parental HeLa/Vero E6 and the corresponding DENV1-4 prME expressing cells were pre-seeding in 96 well plates. After overnight incubation, 50mM SU6656 stock was diluted in cell culture medium to a final concentration as indicated in figures and added to cells. For 0µM control, same volume of DMSO as that in the SU6656 treated group was added to cells. After a day of incubation, cell culture medium was replaced with 200µl 0.8mg/ml MTT solution (Sigma) followed by incubation for 4hrs. Finally, the formazan crystals formed by the MTT reagent was dissolved in 100 µl of isopropanol (Merck) and incubated at 4ºC for 30 minutes of shaking. The percentage of viable cells was calculated by comparing the absorbance of SU6656 treated groups to the 0µM control group at 570 nm.

2-Bromopalmitate treatment to inhibit palmitoylation

Each 60-mm plate of VLP-secreting cells was starved for 1 h in DMEM containing 2.5% FBS and 0.5% defatted BSA with or without indicated concentrations of 2-bromopalmitate. After replacing with fresh media and overnight culture at 37 °C, the VLPs were collected from supernatants, concentrated by ultracentrifugation, subjected to SDS-PAGE, and immunoblotted with anti-E 4G2 antibodies.

siRNA depletions of SFKs

Src siRNA (L-003175), Fyn siRNA (L-003140) and Lyn siRNA (L-003153) were provided as SMARTpool ON-TARGET plus siRNAs, which are pools of four siRNAs targeting various sites in a single gene. For siRNA experiments, reverse transfection was performed using DharmaFECT1 reagents as recommended by the manufacturer. Briefly, siRNAs mixed with DharmaFECT1 reagents were added to 24-well plates in

DMEM medium without FBS and antibiotics. Twenty minutes later 0.8 ml cells (60,000 cells/ml in DMEM supplemented with 10% FBS) were added to each well to final siRNA concentrations. Cells were then incubated at 37°C for 72 hours. For VLP assays, medium was replaced with 0.3 ml of Opti-MEM and, 14 hours later, culture supernatant containing secreted VLPs was collected and clarified by centrifugation at 4,000 rpm for 5 min. Cells separated from supernatants were lysed in RIPA buffer containing freshly added protease inhibitors cocktail (Roche), for 30 min on ice.

Lck inducible expression

Lentiviral particles were produced in HEK293T cells by co-transfection of Lck lentiviral expression vectors with the packaging plasmids as described in the materials and methods. Viral supernatants were collected after 48 h, filtered and used for transduction of cells with 5 μg/ml Polybrene. Puromycin selection was applied at 1 μg/ml for Lck expression.

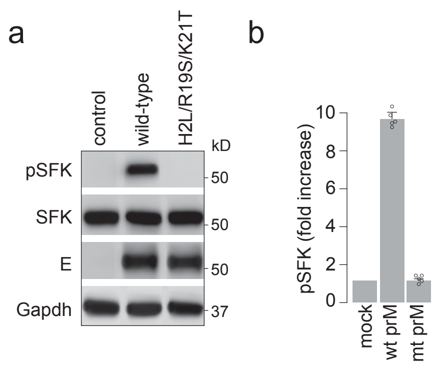

Figure S1. KDELR-prM interaction activates SFKs

a. Cells constitutively secreting Dengue VLPs generated from either wild-type prME or prME carrying three mutations (H2L/R19S/K21T) blocking interaction with KDELR, were immunoblotted to detect phosphorylated SFKs and total SFK **b.** Densitometric analyses were performed on immunoblots from 5 independent experiments. Data are presented as fold change of pSFK/total SFK for each sample compared to control cells set at 1. Error bars represent mean±s.d from 5 independent experiments.

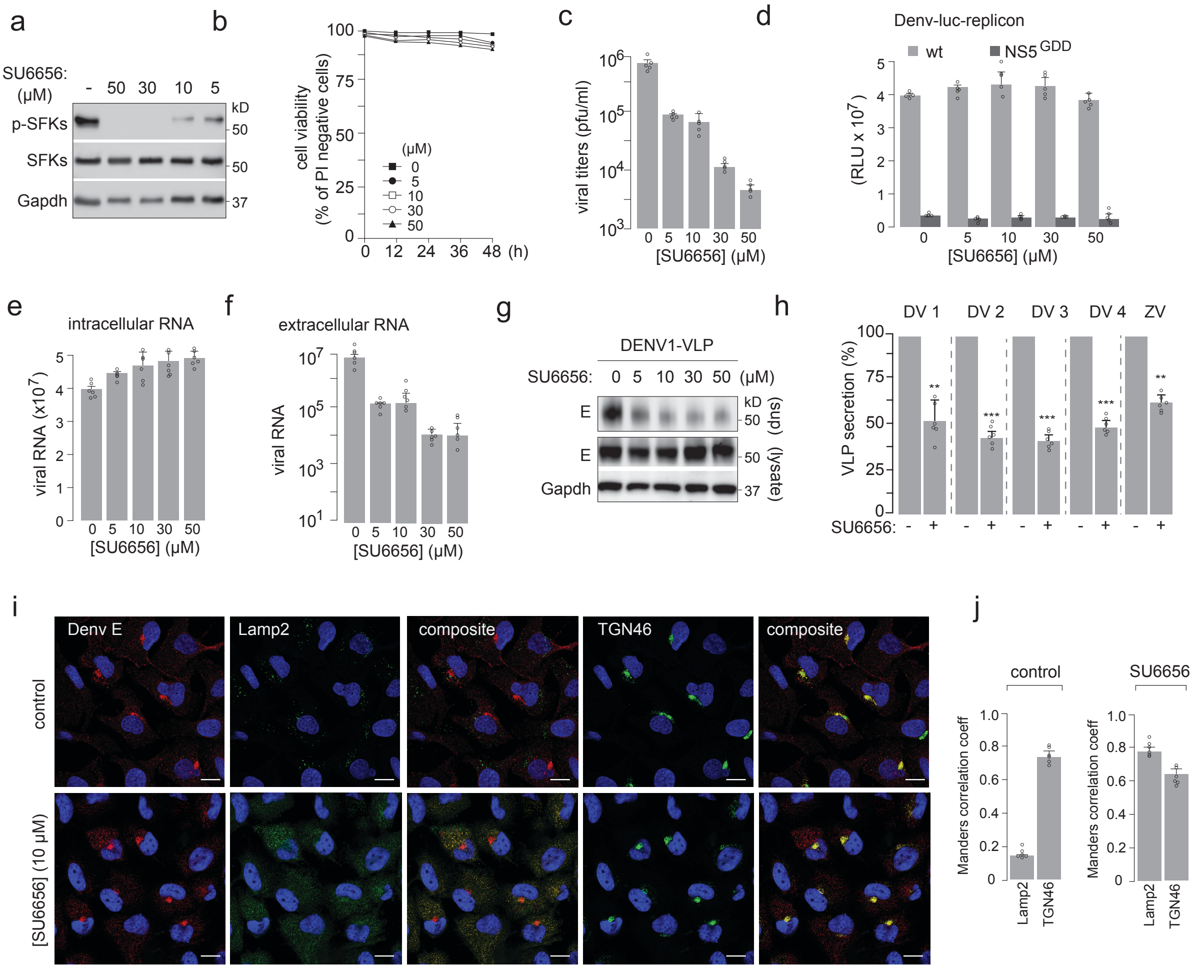

Figure S2: Pharmacological inhibition of SFKs blocks Dengue virus secretion

a. Huh7 cells were treated with different concentrations of a selective SFK inhibitor (SU6656); phosphorylation of the activation loop was measured by immunblotting with anti-phospho-SFK antibodies. Total SFKs were detected using anti-Src rabbit polyclonal antibodies cross-reactive against all SFK members. Gapdh was used as loading control **b.** Huh7 cells were treated with varying concentrations of the selective SFK inhibitor SU6656 and incubated for indicated time intervals at 37ºC, following which, culture medium was replaced with 200μl 0.8mg/ml MTT solution and incubated for 4h. Formazan crystals formed by the MTT reagent were dissolved in 100 μl of isopropanol and incubated at 4ºC for 30 minutes of shaking. The percentage of viable cells was calculated by comparing the absorbance of SU6656 treated groups to the 0μM control group (DMSO only) at 570 nm **c.** Cells treated with SU6656 were challenged with Dengue virus (DENV 2) at MOI 2. 36 hours post infection, supernatants from infected cells were measured for production of virus particles using plaque assay. Error bars represent mean±s.d, n=5 **d.** Cells were transfected with a Denv2 replicon carrying either an active or dead NS5 polymerase gene and treated with SU6656 inhibitor as described in c, to measure luciferase activity (mean±s.d; n=5) **e, f.** intra and extracellular viral RNA was measured using RT qPCR (mean±s.d; n=6) **g.** Secretion of Dengue VLPs from cells treated with varying concentrations of SU6656 was measured by immunoblotting with anti-Dengue 4G2 antibodies, in supernatants and cell lysates. Gapdh was used as loading control **h.** Quantitation of VLP secretion described in **g** by densitometric analyses for all serotypes of Dengue and Zika virus.

Percentage VLP secreted was calculated as fraction of total VLPs normalised to untreated cells set at 100%. Data are presented as a percentage of the control (untreated) value (mean ± s.d; n=6). **$P < 0.01$; ***$P < 0.001$ (compared with vehicle treatment by ANOVA followed by one-sided Dunnett's test) **i.** Confocal imaging was performed to visualise distribution of E-protein in untreated and SU6656 treated cells. Lamp2 and TGN46 were taken as lysosome and Golgi markers **j.** Manders correlation coefficients were measured to quantitate colocalization of E with the organelle markers. Data are presented as mean ± s.e.m; n=6)

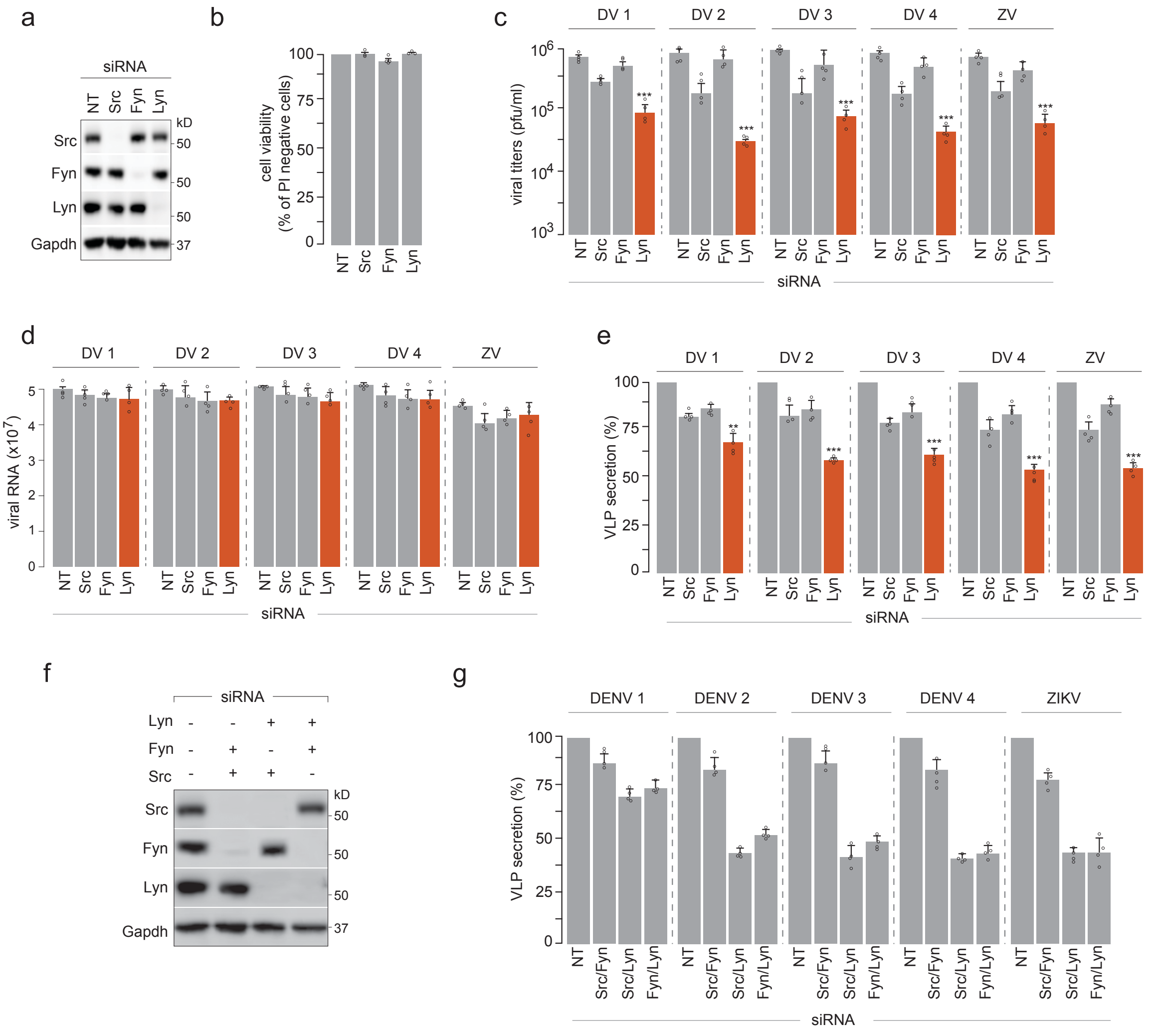

Figure S3: Genetic depletion of SFKs blocks Dengue virus secretion

a. Depletions of Lyn, Fyn and Src were performed in Huh7 cells by siRNA targeting individual kinases, and verified by immunoblotting with kinase specific antibodies **b.** Cell viability was measured for all cells with 200μl 0.8mg/ml MTT solution. The percentage of viable cells was calculated by comparing the absorbance of non-targeting siRNA-treated cells at 570 nm (mean±s.d; n=3) **c, d.** SFK-depleted cells were challenged with different serotypes of Dengue virus and Zika. Viral titers were measured in supernatants collected from infected cells, using plaque assays (c); Error bars represent mean±s.d; n=4; ***$P < 0.001$ (compared with control by ANOVA followed by one-sided Dunnett's test) and qPCR (d); error bars represent mean±s.d; n=4 **e.** Depletion of SFKs was performed in the corresponding VLP-producing cells to measure secretion, using 4G2 antibodies to detect viral structural proteins as readout. Percentage VLP secreted was calculated as fraction of total intracellular VLPs normalised to control (NT siRNA-treated) cells set at 100%. Data are presented as a percentage of the control (NT siRNA-treated) value (mean ± s.d of 4 independent experiments); **$P < 0.01$; ***$P < 0.001$ (compared with NT control by ANOVA followed by one-sided Dunnett's test) **f.** Combinations of Lyn, Fyn and Src depletions were performed by co-transfections of siRNA targeting individual kinases in VLP-producing cells, and verified by immunoblotting with specific antibodies **g.** VLP secretion from non-targeting and siRNA-treated cells was measured using 4G2 antibodies to detect viral structural proteins as readout. Quantitation of VLP secretion was performed by densitometric analyses for all serotypes of Dengue and Zika virus. Percentage

secreted was calculated as fraction of total intracellular VLPs normalised to untreated cells set at 100%. Error bars represent mean±s.d from 4 independent biological replicates.

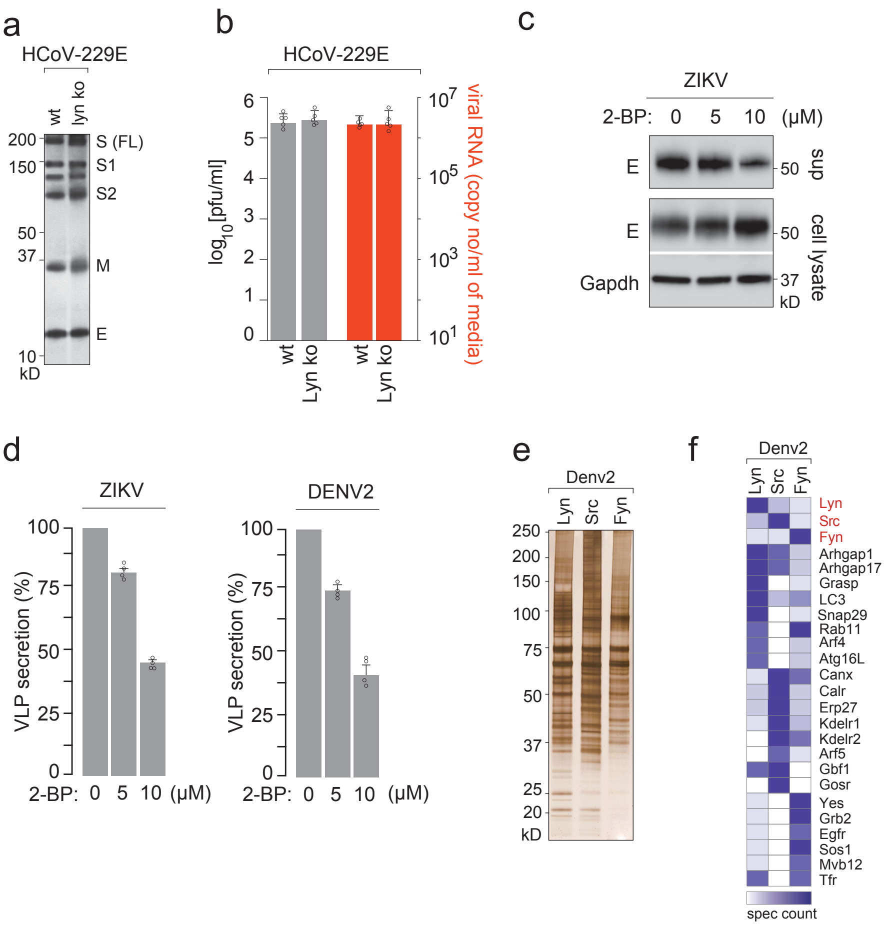

Figure S4: Characterization of Lyn in virus secretion

a. Huh7 cells (wt and Lyn$^{-/-}$) were infected with human coronavirus (HCoV-229E; MOI 5; 48 h). Cells were metabolically labelled with [^{35}S]cys/met; supernatants collected were resolved by SDS-PAGE and secreted particles were detected by autoradiography **b.** Supernatants were also collected to measure viral titres using plaque assay (grey bars) and viral RNA using RT-qPCR (red bars). Data represent mean ± s.d; n=5 **c.** VLP-producing cells were treated with 0, 5 or 10 μM 2-BP and cultured overnight at 37°C. Secreted VLPs were collected from supernatants, concentrated, resolved by SDS-PAGE and detected by anti-E 4G2 antibodies. Corresponding lysates were prepared to measure total intracellular VLPs by gel electrophoresis and detection by immunoblotting using 4G2 antibodies. Gapdh levels were measured as loading controls **d.** Quantitation of VLP secretion was performed by densitometric analyses for Dengue 2 and Zika virus. Percentage VLP secreted was calculated as fraction of total intracellular VLPs normalised to untreated cells set at 100%. Error bars represent mean±s.d from 4 independent biological replicates **e.** Immunoprecipitation of Lyn, Src and Fyn were performed from Dengue-infected cells on individual antibodies. Co-precipitating material was resolved by SDS-PAGE and visualized by silver staining **f.** Whole lanes on gels were sliced into 2 mm sections and subjected to trypsin digest. The peptide mix was processed and analysed by an LTQ Orbitrap mass spectrometer. MS/MS spectra were analyzed using Sequest algorithm searching a composite target-decoy protein sequence database. The target sequences comprised the human protein repository of the Uniprot database. Spectral counts for each sample were combined at a protein level and normalized by protein length to infer protein abundances and intensities in each case. Identified hits were further categorized into different biological pathways using Gene Ontology analyses and Ingenuity Pathway Analyses software.

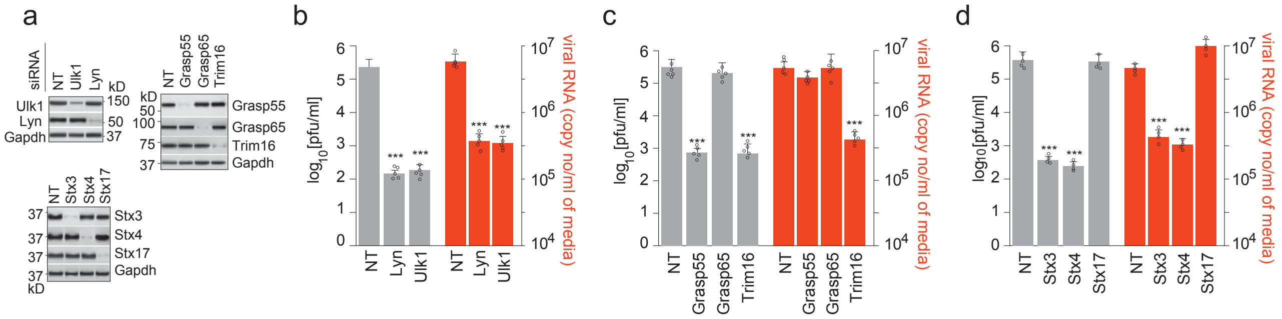

Figure S5: Validation of genes implicated in secretory autophagy in Dengue-infected monocytes

a. Depletion of indicated proteins were performed by siRNA transfections in human CD14+ monocytes, and verified by immunoblotting **b-d.** Cells described in **a** were infected with Zika at MOI 2 for 48h and measured for infectivity using plaque assays (grey bars) and extracellular vRNA using RT qPCR (red bars). Data represent mean ± s.d of 5 biological replicates. ****P* < 0.001 (compared with NT control by ANOVA followed by one-sided Dunnett's test).

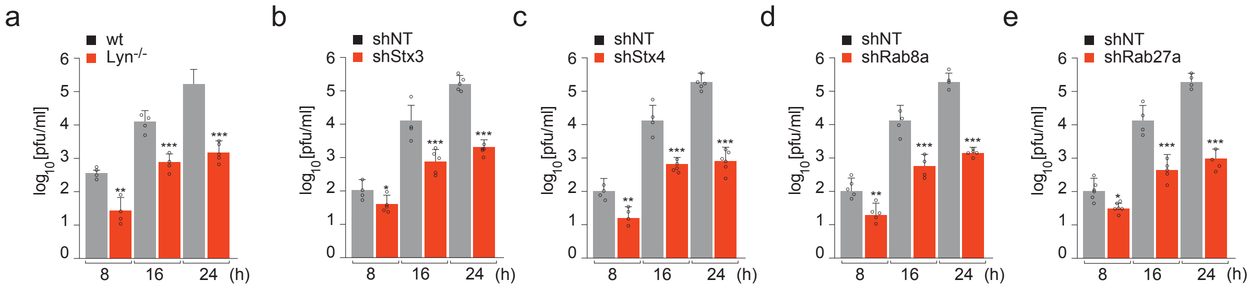

Figure S6: Genetic validation of Lyn kinase and genes of the secretory autophagy pathway in Dengue virus production

Huh7 cells which are Lyn-deficient **(a)** or those depleted in Stx3 **(b)**, Stx4 **(c)**, Rab8a **(d)** and Rab27a **(e)** described in Fig 7h were cultured in medium supplemented with 10 mM BSA-conjugated fatty acids and infected with Dengue virus at MOI 1 for 8, 16 and 24h and measured for infectivity using plaque assays. Data represent mean ± s.d; n=4 or 5 as indicated. $^{**}P < 0.01$; $^{***}P < 0.001$ (compared with NT control by ANOVA followed by one-sided Dunnett's test).

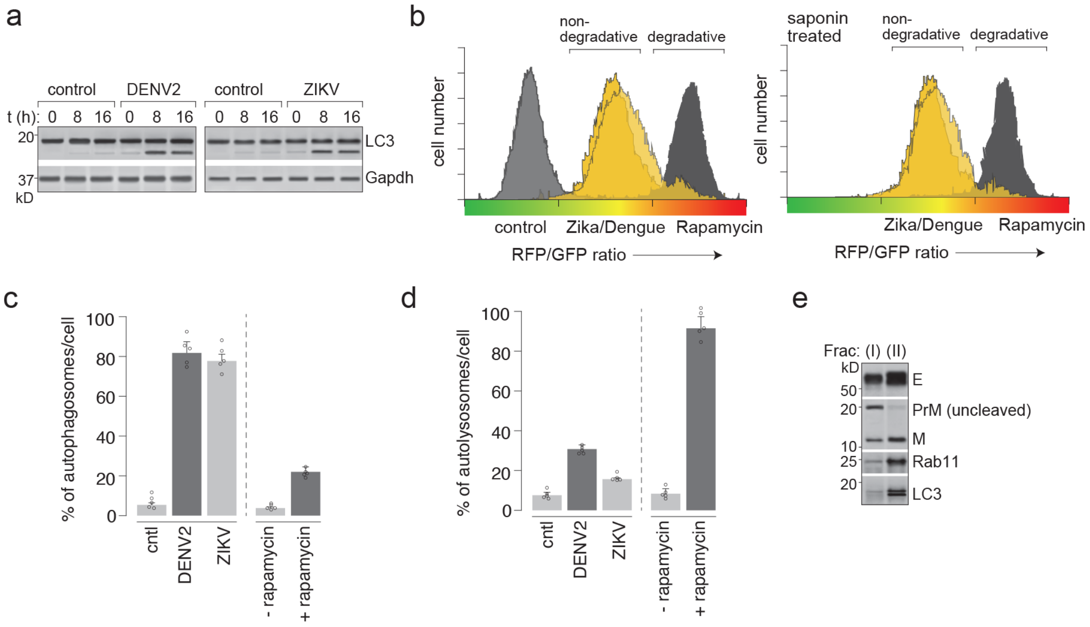

Figure S7: Generation of non-degradative autophagosomes in flavivirus infections

a. Control (mock-infected) and either Dengue or Zika virus-infected cells (MOI 2) at indicated time intervals were immunoblotted for LC3-I/II **b.** (*Left panel*) cells stably expressing GFP-RFP-LC3 reporter for measuring autophagic flux, to distinguish between formation of secretory versus degradative autophagosomes were infected with either Dengue or Zika virus (MOI 2; 18h; ~80% infected cells as measured by viral E protein+ fraction in the total population). Control cells for degradative lysosomes were cultured in media±rapamycin to induce formation of degradative autolysosomes as controls. (*Right panel*) Dengue/Zika infected cells were treated with saponin to first remove non-lipidated LC3 before analyses by flow cytometry. Ratiometric flow-cytometry analysis (RFP/GFP) was performed to measure % of non-degradative versus degradative autophagosomes in either the virus-infected/rapamycin-treated population. **c, d.** Formation of autophagosomes and autolysosomes was quantitated by RFP/GFP fluorescence ratio and presented as % of total (as measured by total LC3+ signal); non-degradative autophagosomes are represented by yellow fluorescence signal whereas autolysosomes by red fluorescence. Error bars were calculated as mean±sd; n=5 **e.** Extracellular virus populations separated over sucrose gradients (described in Fig 7) were concentrated and immunoblotted for viral structural proteins E, prM and also LC3 and Rab11.

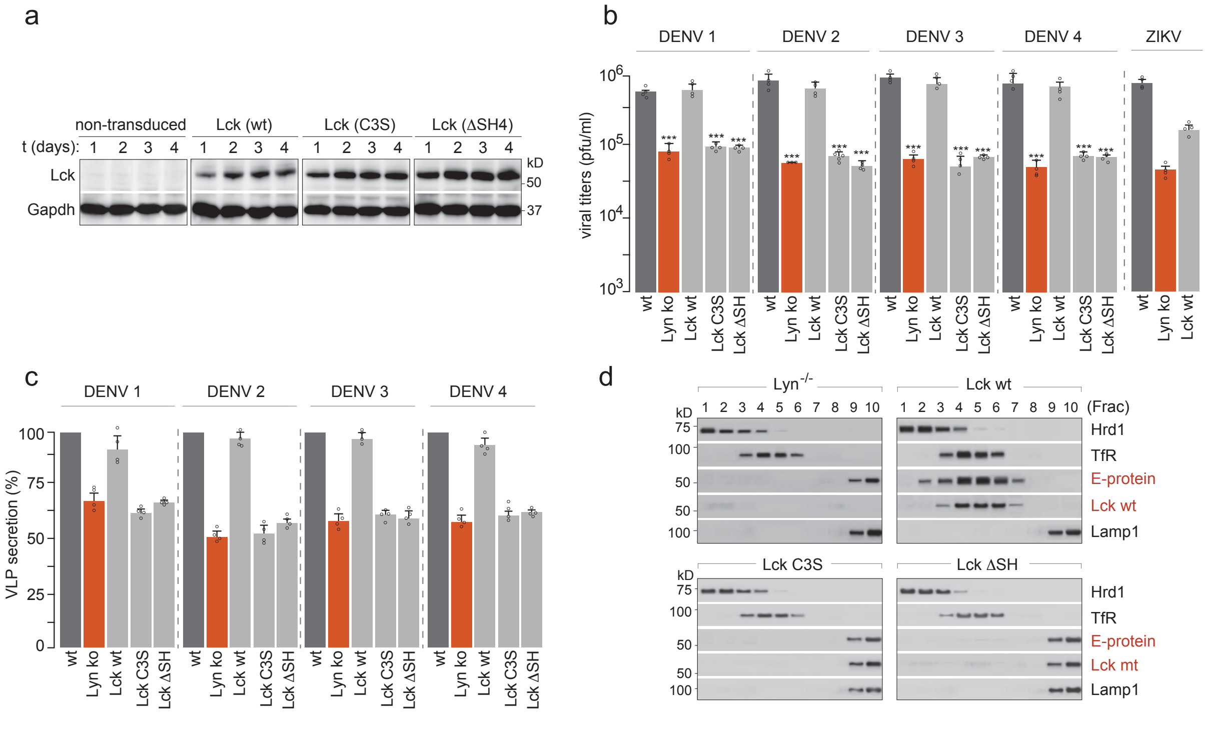

Figure S8. Lyn-dependent Dengue virus transport from the Golgi can be rescued by expressing Lck

a. Inducible expression of wild-type, palmitoylation-deficient (C3S) and SH4-domain truncation (ΔSH4) mutants of Lck in the $Lyn^{-/-}$ cells. Validation of Lck expression was performed upto 4 days post induction by immunoblotting with Lck-specific antibodies. **b.** Lck-expressing cells described in (a) were challenged with Dengue and Zika at MOI 2,. Supernatants collected from infected cells after 48 h were tested for viral titres using plaque assays. Error bars represent mean±s.d from 4 biological replicates; *** represents p<0.001 (compared with wild-type by ANOVA followed by one-sided Dunnett's test). **c.** VLP-secretion was measured in Lck-expressing cells by immunoblotting for viral structural proteins in supernatant preparations. Percentage VLP secreted was calculated as fraction of total intracellular VLPs normalised to untreated cells set at 100%. Error bars represent mean±s.d from 4 independent biological replicates **d.** Subcellular distribution of viral E-protein in membrane fractions were generated from Lck-expressing cells by biochemical separations described in figure 5b.

SI Table S1 qPCR primers

Name	Forward primer	Reverse primer
DENV-2	5’-gcccttctgttcacaccatt-3’	5’-ccacatttgggcgtaagact-3’
DENV-universal	5’-ggttagaggagacccctccc-3’	5’-ggtctcctctaacctctagtcc-3’
ZIKV	5’-agatgaactgattggccgggc-3’	5'-aggtctcttctgtggaaata-3'

SI Table S2 Guide RNA for knock-out cells

Name	Expression construct	Guide RNA
LYN	pLynKO-1	5’-TGAAAGACAAGTCGTCCGGG-3’
LYN	pLynKO-2	5’-GTAGCCTTGTACCCCTATGA-3’

SI Table S3 siRNA constructs

Gene	Accession Number	siRNA (SMARTpool)
Human LYN	NM_002350	L-003153
Human FYN	NM_153047.4	L-003140
Human SRC	NM_198291	A-003175
Non targeting		A-001206

SI Table S4 shRNA constructs

Name	Accession Number	clone/lentiviral particles
GORASP2	NM_015530	TRCN0000278363
GORASP1	NM_031899	TRCN0000155672
STX3	NM_004177	sc-41328-V
STX4	NM_004604	sc-36590-V
STX17	NM_017919	sc-92492-V
TRIM16	NM_006470	sc-76740-V
ULK1	NM_003565	sc-44182-V
FIP200	NM_014781	sc-38211-V
ATG13	NM_001346312.1	sc-97013-V
RAB8A	NM_005370	sc-41828-V
RAB27A	NM_183236	sc-41834-V
RAB11A	NM_004663	sc-36340-V
RAB7	NM_004637	sc-29460-V
