# Supplementary material for: Lyn kinase regulates egress of flaviviruses in autophagosome-derived organelles
Source: Nat Commun. 2020 Oct 15;11:5189. doi: 10.1038/s41467-020-19028-w (PMC7564011; doi:10.1038/s41467-020-19028-w)
Supplement: Supplementary file 3 — Reporting Summary [file 41467_2020_19028_MOESM3_ESM.pdf]

nature research

Corresponding author(s): Sumana Sanyal

Last updated by author(s): 2020-Aug-07

Reporting Summary

Nature Research wishes to improve the reproducibility of the work that we publish. This form provides structure for consistency and transparency in reporting. For further information on Nature Research policies, see Authors & Referees and the Editorial Policy Checklist .

Statistics

For all statistical analyses, confirm that the following items are present in the figure legend, table legend, main text, or Methods section.

n/a	Confirmed	
☐	☒	The exact sample size (*n*) for each experimental group/condition, given as a discrete number and unit of measurement
☐	☒	A statement on whether measurements were taken from distinct samples or whether the same sample was measured repeatedly
☐	☒	The statistical test(s) used AND whether they are one- or two-sided<br>*Only common tests should be described solely by name; describe more complex techniques in the Methods section.*
☐	☒	A description of all covariates tested
☐	☒	A description of any assumptions or corrections, such as tests of normality and adjustment for multiple comparisons
☐	☒	A full description of the statistical parameters including central tendency (e.g. means) or other basic estimates (e.g. regression coefficient) AND variation (e.g. standard deviation) or associated estimates of uncertainty (e.g. confidence intervals)
☐	☒	For null hypothesis testing, the test statistic (e.g. *F*, *t*, *r*) with confidence intervals, effect sizes, degrees of freedom and *P* value noted<br>*Give P values as exact values whenever suitable.*
☒	☐	For Bayesian analysis, information on the choice of priors and Markov chain Monte Carlo settings
☒	☐	For hierarchical and complex designs, identification of the appropriate level for tests and full reporting of outcomes
☐	☒	Estimates of effect sizes (e.g. Cohen's *d*, Pearson's *r*), indicating how they were calculated

Our web collection on statistics for biologists contains articles on many of the points above.

Software and code

Policy information about availability of computer code

Data collection	Confocal images were obtained with a either a Zeiss Observer Z.1 or an LSM 700 confocal microscope using the Zeiss ZEN software.
Data analysis	ImageJ and FIJI (version 2.0.0-rc-69/1.52p) were used for densitometric and confocal image analyses. Ingenuity Pathway Analyses and Panther were used for performing gene ontology analyses. GraphPad Prism version 8 for statistical analyses, Imaris version 9.2 for colocalizations, SEQUEST HT for database search.

For manuscripts utilizing custom algorithms or software that are central to the research but not yet described in published literature, software must be made available to editors/reviewers. We strongly encourage code deposition in a community repository (e.g. GitHub). See the Nature Research guidelines for submitting code & software for further information.

Data

Policy information about availability of data

All manuscripts must include a data availability statement. This statement should provide the following information, where applicable:

- Accession codes, unique identifiers, or web links for publicly available datasets
- A list of figures that have associated raw data
- A description of any restrictions on data availability

Protein sequences used in this study were extracted from UniprotKB (Human; release 2020_06 including isoforms and unreviewed sequences; https://www.uniprot.org/uniprot/). Protein sequences of Dengue and Zika strains were extracted from UniprotKB. All accession codes of RNAi experiments have been provided in the supplementary information. Source files for immunoblots presented in the manuscript have been provided. The remainder of the data generated or analysed during the current study are available from the corresponding author on reasonable request.

Field-specific reporting

Please select the one below that is the best fit for your research. If you are not sure, read the appropriate sections before making your selection.

[x] Life sciences [ ] Behavioural & social sciences [ ] Ecological, evolutionary & environmental sciences

For a reference copy of the document with all sections, see nature.com/documents/nr-reporting-summary-flat.pdf

Life sciences study design

All studies must disclose on these points even when the disclosure is negative.

Sample size	Samples sizes were selected keeping in mind the variability between independent sources of cells. All experiments were performed in mammalian cell cultures, which are population-based, with data points generated from experiments performed from cells generated from independent clones and performed independent of each other.
Data exclusions	No data was excluded.
Replication	For all experiments data were generated with atleast three independent biological replicates along with atleast 2 technical replicates to calculate the mean and standard deviation. All attempts at replication were successful.
Randomization	Cells from different clones were independently replicated on atleast 3 separate days, with wells in microtiter plates randomised for treatment conditions, and measurements taken on ~1 x E6 cells from each well.
Blinding	No blinding was performed.

Reporting for specific materials, systems and methods

We require information from authors about some types of materials, experimental systems and methods used in many studies. Here, indicate whether each material, system or method listed is relevant to your study. If you are not sure if a list item applies to your research, read the appropriate section before selecting a response.

Materials & experimental systems

n/a	Involved in the study
[ ]	[x] Antibodies
[ ]	[x] Eukaryotic cell lines
[x]	[ ] Palaeontology
[x]	[ ] Animals and other organisms
[x]	[ ] Human research participants
[x]	[ ] Clinical data

Methods

n/a	Involved in the study
[x]	[ ] ChIP-seq
[ ]	[x] Flow cytometry
[x]	[ ] MRI-based neuroimaging

Antibodies

Antibodies used	conjugated anti-phospho tyrosine antibodies (Cell Signaling Technology, Cat# 9411); anti-E 4G2 (clone D1-4G2-4-15, Novus Biologicals; Cat# 52709); mouse anti-E 4G2 (homemade mouse ascites using hybridoma cells D1-4G2-4-15 from ATCC (Cat No. BH-112); anti-Phospho-Src Family (Try 416) (clone no D49G4, Cell Signaling, Cat no 694 ; rabbit anti-Lyn mAb (Cat no 2796, clone no C13F9, Cell Signaling); rabbit anti-Src mAb (Cat no 2123, clone no 32G6, Cell Signaling Technology); rabbit anti-Fyn - (Cat no 4023, Cell Signaling); Phospho-Tyrosine Mouse mAb(P-Try-100) conjugated magnetic beads - Phospho-Tyrosine Mouse mAb (P-Tyr-100), Cat no 8095, Cell Signaling, mouse anti-GAPDH mAb - Anti-GAPDH antibody [6C5], Cat no ab8245, Clone no 6C5, Abcam
Validation	anti-E 4G2 (clone D1-4G2-4-15, Novus Biologicals; Cat# 52709) binds to flavivirus group antigen, protein E. Western blotting performed detects the target (band between 55-72kDa). Validated in 3 species: Human, Mouse, Virus for WB, ELISA, ICC/IF.<br>Phospho-Src Family (Tyr416) (D49G4) Rabbit mAb detects endogenous levels of Src only when phosphorylated at Tyr416. Species Reactivity: Human, Mouse, Rat, Monkey. Lyn (C13F9) Rabbit mAb detects endogenous levels of total Lyn protein. This antibody does not cross-react with any other Src-family members. Species Reactivity: Human, Mouse, Rat, Monkey.<br>Fyn Antibody detects endogenous levels of total Fyn proteins. This antibody does not cross-react with Src and Hck family members. Species Reactivity: Human, Mouse. Phospho-Tyrosine Mouse mAb (P-Tyr-100) is a high affinity antibody. ELISAs against a wide variety of phosphopeptides indicate that P-Tyr-100 binds phospho-Tyr in a manner largely independent of the surrounding amino acid sequence. 2D gel Western blot analysis of pervanadate-treated cell extracts also shows that P-Tyr-100 interacts with a broad range of tyrosine-phosphorylated proteins. P-Tyr-100 does not cross-react with peptides containing phospho-Ser or phospho-Thr. Species Reactivity: All Species Expected.<br><br>All antibodies that were purchased from commercial vendors have been validated in different cell types based on their molecular weights and their expression profiles in wild-type and the corresponding knock-down/knock-out models. These data are available

upon request. In-house generated antibodies and other routine antibodies have been previously described in the following manuscripts:

1. Zhang, J., Lan, Y., Li, M. Y., Lamers, M. M., Fusade-Boyer, M., Klemm, E., Thiele, C., Ashour, J., and Sanyal, S. (2018) Flaviviruses Exploit the Lipid Droplet Protein AUP1 to Trigger Lipophagy and Drive Virus Production. Cell Host Microbe. 23, 819–831.e5

2. Li, M. Y., Grandadam, M., Kwok, K., Lagache, T., Siu, Y. L., Zhang, J. S., Sayteng, K., Kudelko, M., Qin, C. F., Olivo-Marin, J.-C., Bruzzone, R., and Wang, P.-G. (2015) KDEL Receptors Assist Dengue Virus Exit from the Endoplasmic Reticulum. CellReports. 10, 1496–1507

3. Sanyal S, Ashour J, Maruyama T, Bilate A, Avalos AM, Kundrat L, Altenburg A, Cragnolini JJ, Garcia-Sastre A and Ploegh H. (2013) Type-I interferon imposes a Tsg101/ISG15 checkpoint at the Golgi for glycoprotein trafficking during influenza infection Cell Host and Microbe, 14(5): 510-521

Eukaryotic cell lines

Policy information about cell lines

Cell line source(s)	Vero E6 - VERO C1008, clone E6, Cat no ATCC® CRL-1586™, BHK-21 [C-13], Cat no ATCC® CCL-10™, Hela cells, Cat no ATCC®, CCL-2™, HeLa LC3 reporter cells, HeLa-Difluo™ hLC3 cells, Cat no: heldf-hlc3 (Invivogen), Hep G2 [HEPG2], Cat no ATCC® HB-8065™, Huh7, Accegen® Cat no ABC-TC0437, 293T cells, Cat no ATCC® CRL-3216™.
Authentication	All cell lines were purchased from either ATCC or Invivogen, which were characterized and authenticated by short tandem repeat (STR) DNA profiles.
Mycoplasma contamination	all cell lines tested and confirmed to be negative for mycoplasma
Commonly misidentified lines (See ICLAC register)	No commonly misidentified cell lines (reported by ICLAC) have been used in this study.

Flow Cytometry

Plots

Confirm that:

- [x] The axis labels state the marker and fluorochrome used (e.g. CD4-FITC).
- [x] The axis scales are clearly visible. Include numbers along axes only for bottom left plot of group (a 'group' is an analysis of identical markers).
- [x] All plots are contour plots with outliers or pseudocolor plots.
- [x] A numerical value for number of cells or percentage (with statistics) is provided.

Methodology

Sample preparation	At designated time points, the cells were dissociated with 10 mM EDTA in PBS at 37˚C. Cells were then stained with 1:200 Zombie NIR™ Fixable Viability Kits or for 10 to 15 minutes. After that, the unbound dyes were neutralized and washed away with FACS buffer (PBS containing 2% FBS and 0.004% sodium azide [w/v]). Cells were washed with FACS buffer and fixed with 4% formaldehyde diluted in PBS. Cells were then permeabilized using BD Perm/Wash Buffer (BD Biosciecences; 554723) at room temperature for 20 minutes. Intracellular proteins were then stained with 50 µL of 4G2 antibodies diluted with BD Perm/Wash Buffer on ice for 30 minutes. Cells were then washed with BD Perm/Wash Buffer and resuspended in FACS buffer before data acquisition. The GFP-positive cells (GFP) stained with anti-E MAb 4G2 (4G2) were analyzed by flow cytometry.
Instrument	FACS data was acquired on Attune NxT Flow Cytometer.
Software	All FACS data were analysed using FlowJo 10.
Cell population abundance	There is no sorting involved in this study.
Gating strategy	For all the experiments, cells were first gated on single cells by a forward side scatter gate, followed by GFP gating by excluding dead cells. GFP+ and 4G2 populations were gated in consistence with the corresponding negative controls.

- [x] Tick this box to confirm that a figure exemplifying the gating strategy is provided in the Supplementary Information.
