# Supplementary material for: Lyn kinase regulates egress of flaviviruses in autophagosome-derived organelles
Source: Nat Commun. 2020 Oct 15;11:5189. doi: 10.1038/s41467-020-19028-w (PMC7564011; doi:10.1038/s41467-020-19028-w)
Supplement: Supplementary file 4 — Description of Additional Supplementary Files [file 41467_2020_19028_MOESM4_ESM.pdf]

Description of Additional Supplementary Files

File Name: Supplementary Data 1
Description: Raw data for candidates identified by mass spectrometry).
