# Supplementary material for: Lyn kinase regulates egress of flaviviruses in autophagosome-derived organelles
Source: Nat Commun. 2020 Oct 15;11:5189. doi: 10.1038/s41467-020-19028-w (PMC7564011; doi:10.1038/s41467-020-19028-w)
Supplement: Supplementary file 6 — Source Data [file 41467_2020_19028_MOESM6_ESM.zip › Source Data/uncropped blot scans.docx]

**Figure 1a, c**: Antibodies: anti-pSFK; CST Rabbit mAb # 6943, #2101; anti Gapdh; Abcam [6C5]; #Ab8245; anti E mAb 4G2 prepared using hybridoma cells D1-4G2-4-15 (ATCC)


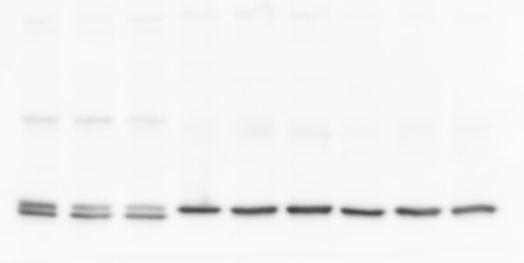


BHK21


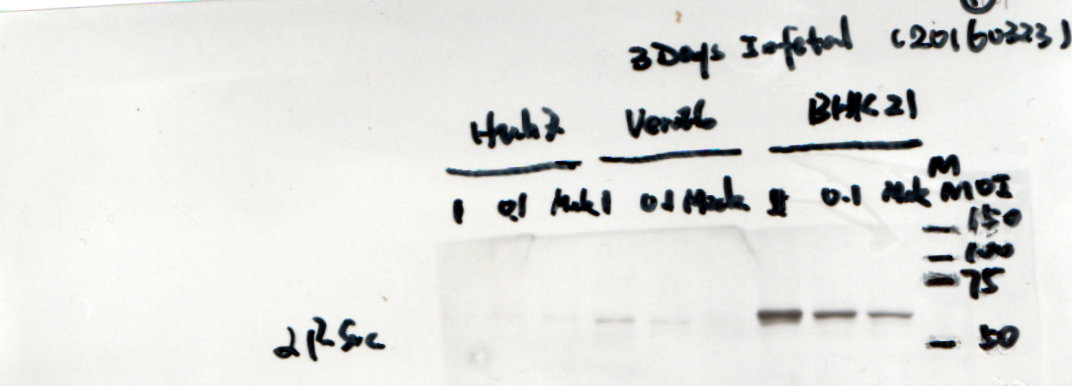


Anti-pSFK anti-Gapdh


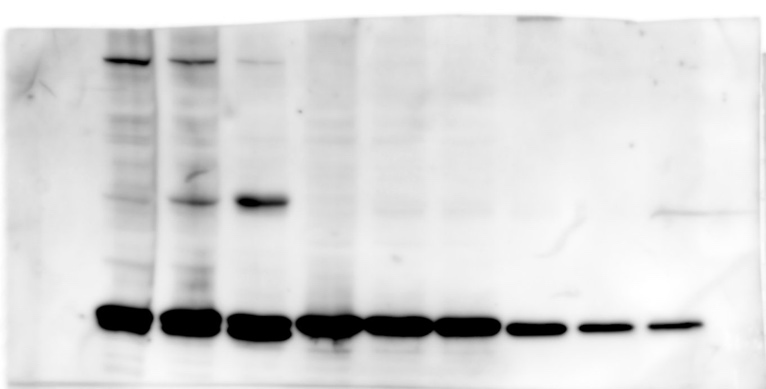


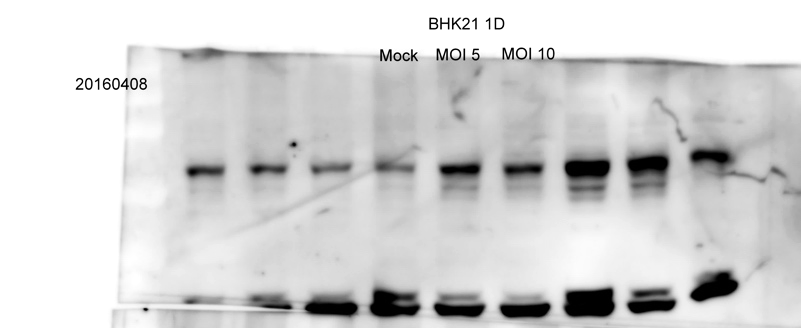


Anti-SFK Anti-pSFK, anti-Gapdh

Vero E6


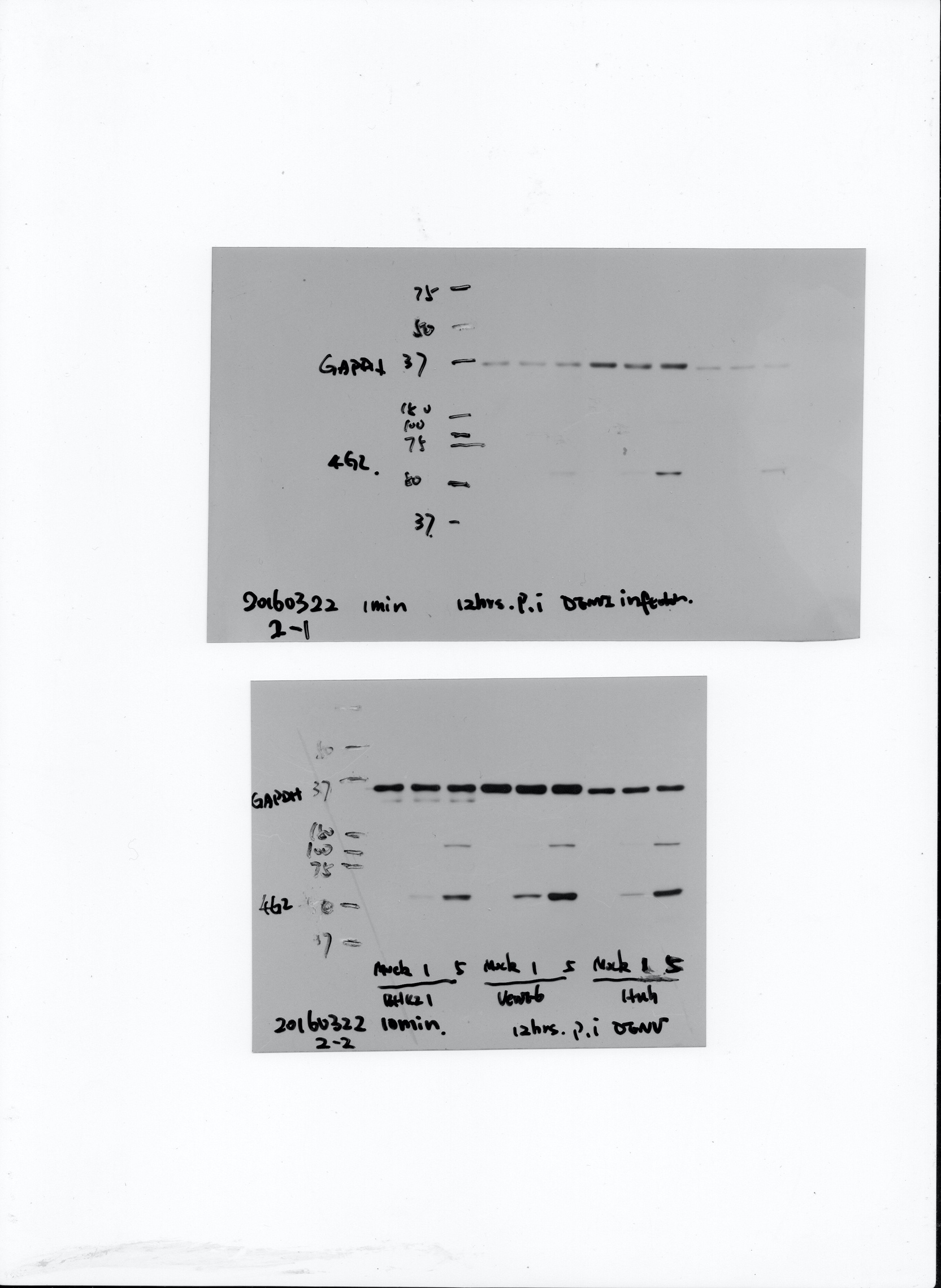


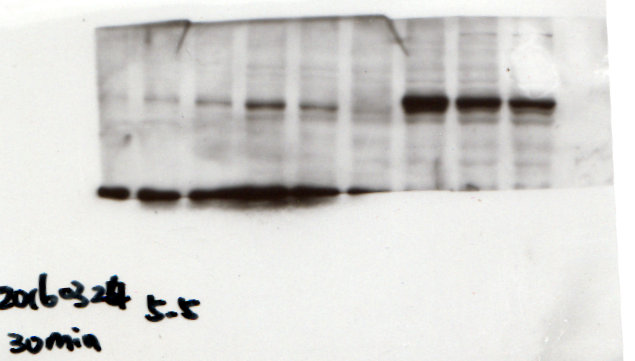


Anti-pSFK anti-Gapdh

Anti-E; BHK21 and Vero E6; anti E mAb 4G2 prepared using hybridoma cells D1-4G2-4-15 (ATCC)


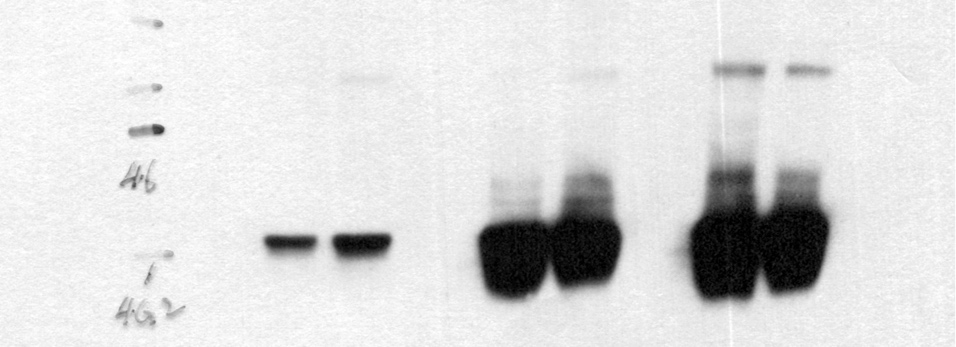


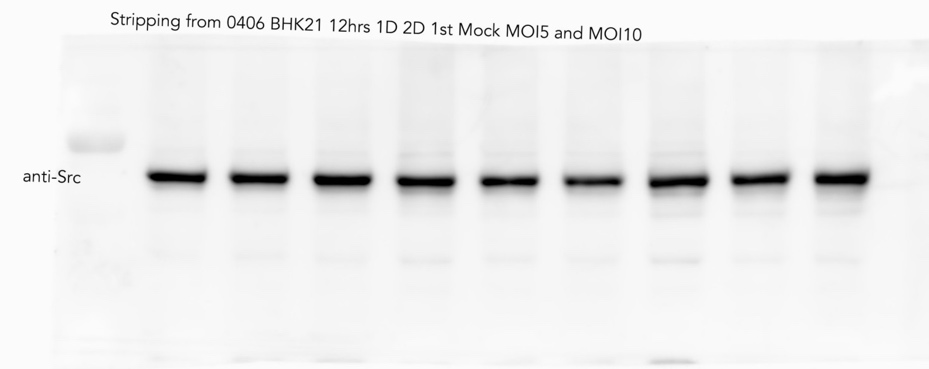
Anti SFKs; CST; #9320

Figure 1c


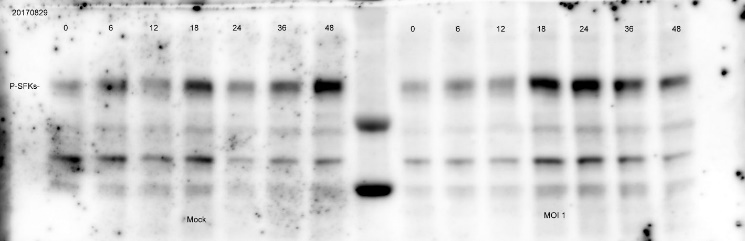


Anti-pSFK


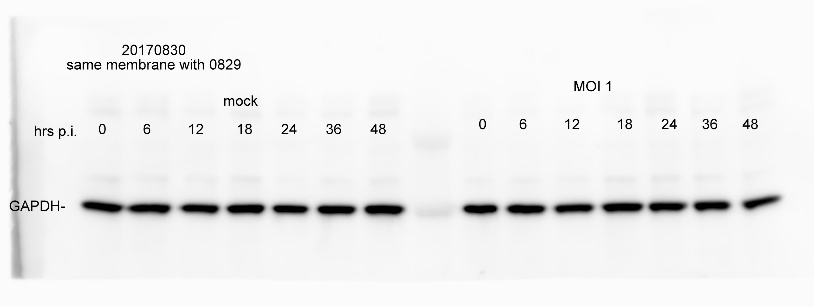

 Anti-Gapdh

Anti-pSFK and anti-Gapdh

**Figure 1e**: Antibodies: anti-pSFK; CST Rabbit mAb # 6943, #2101; anti Gapdh; Abcam [6C5]; #Ab8245; anti E mAb 4G2 prepared using hybridoma cells D1-4G2-4-15 (ATCC)


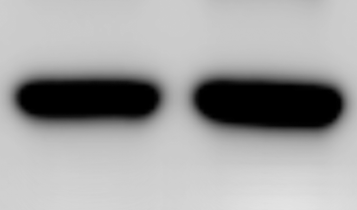

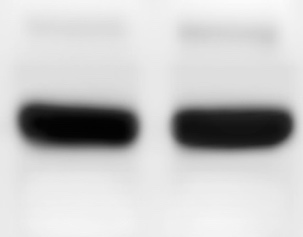

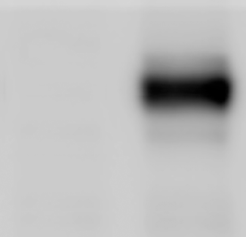

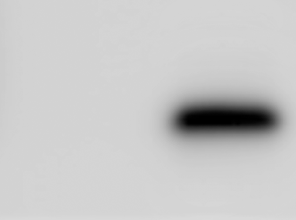


anti-pSFK anti E mAb anti-SFK anti Gapdh


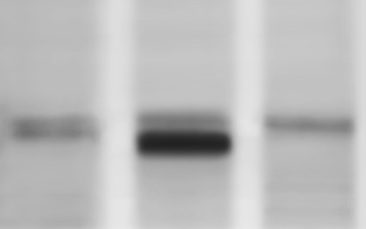

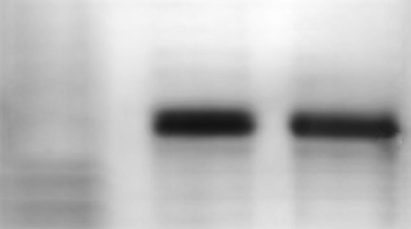

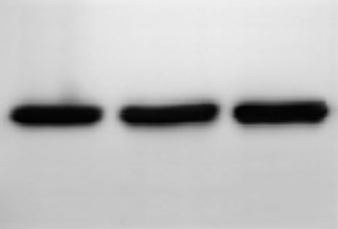

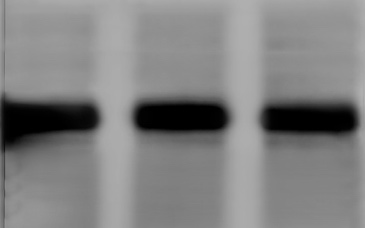
anti-pSFK ssHRP-KDEL anti Gapdh anti-SFK

**
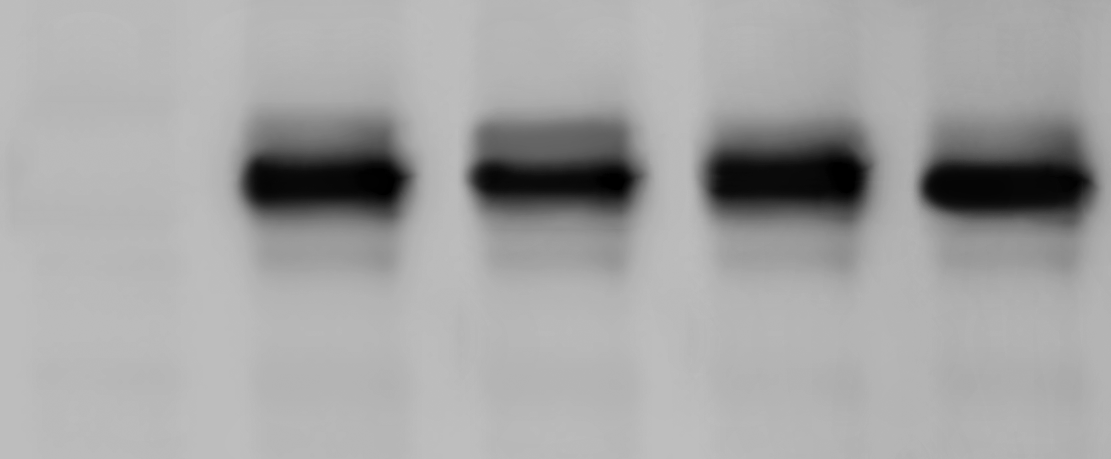
**

**
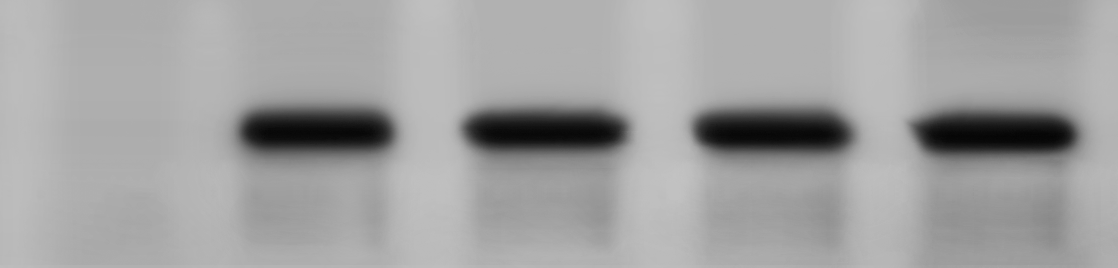
**

anti-pSFK anti E mAb

**
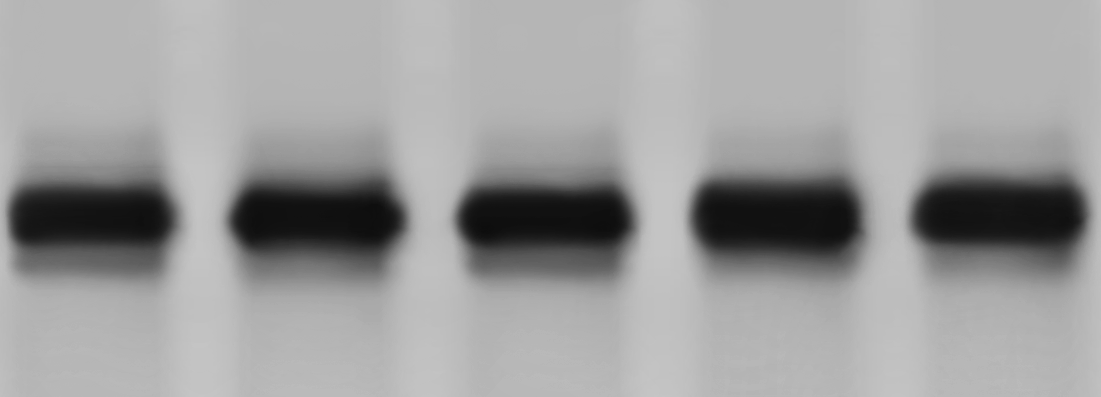

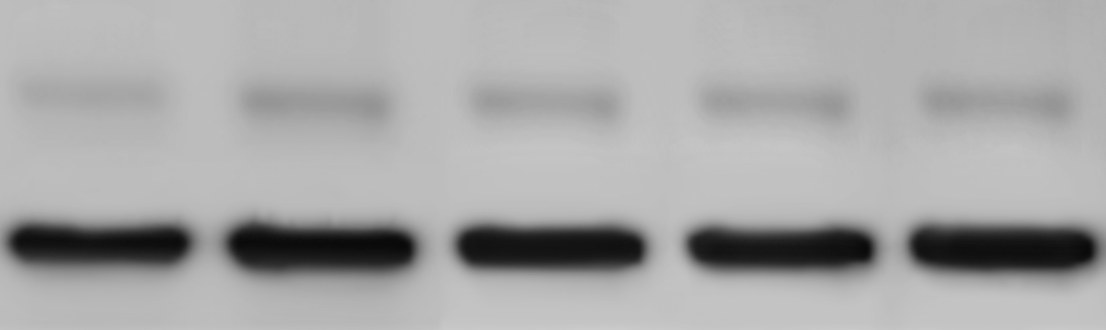
**

anti-Gapdh anti-SFK


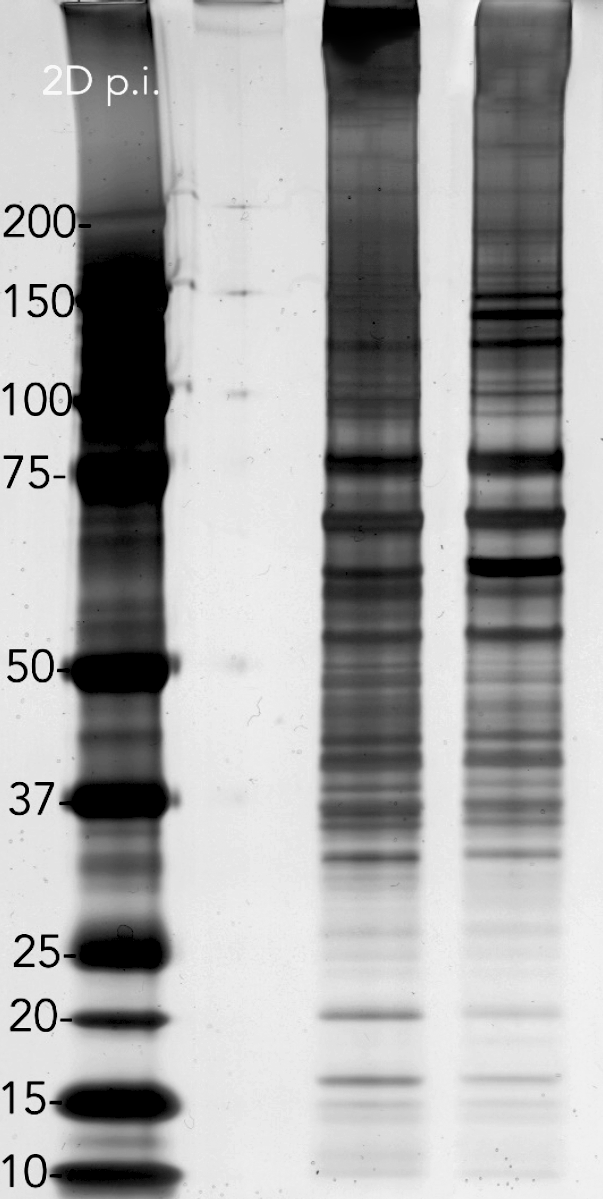
**Figure 2a**: IP on anti-pSFK; CST #2101 **Figure 2d**: IP anti-pTyr; mouse mAb (magnetic bead conjugate #8095); anti-Src; CST; rabbit mAb; 36D10, anti-Fyn; CST; rabbit mAb; #4023; anti-Lyn; CST; rabbit mAb (C13F9); #2796; anti-Lyn; CST; mouse mAb (5G2); #4576, anti- Gapdh; Abcam [6C5]; #Ab8245


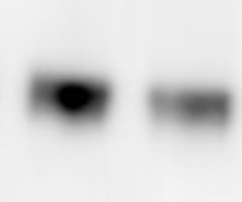

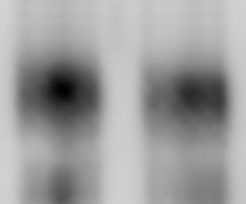

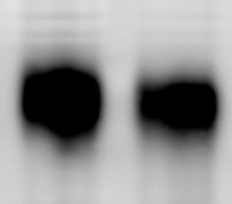


Anti-Src anti-Fyn anti-Lyn


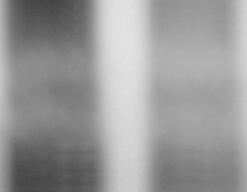

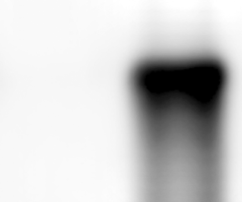


Anti-E anti-E (eluate)


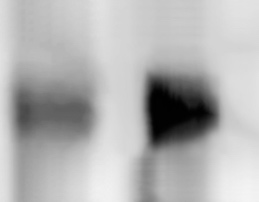

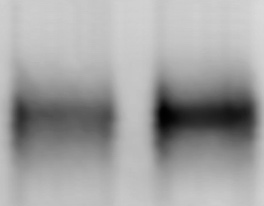

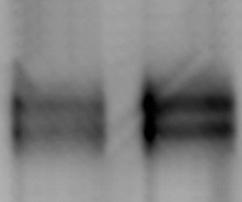


Anti-Src anti-Fyn anti-Lyn

**Figure 2c**: anti-Src; CST; rabbit mAb; 36D10, anti-Fyn; CST; rabbit mAb; #4023; anti-Lyn; CST; rabbit mAb (C13F9); #2796; anti-Lyn; CST; mouse mAb (5G2); #4576, anti- Gapdh; Abcam [6C5]; #Ab8245


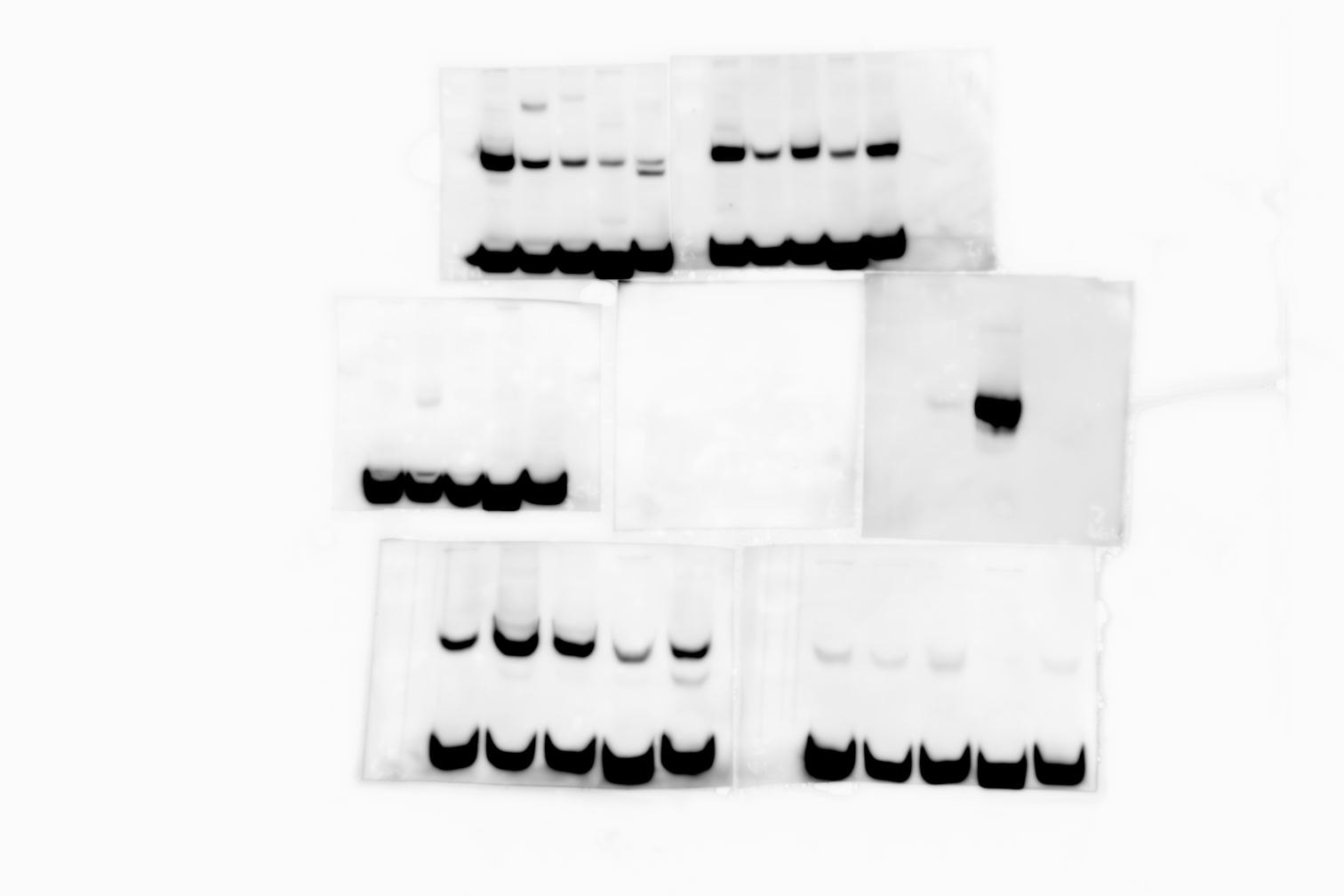


1: HeLa

2: HepG2

3: Huh7

4: BHK21

5: Vero E6

**Figure 2e**: anti-Src; CST; rabbit mAb; 36D10, anti-Fyn; CST; rabbit mAb; #4023; anti-Lyn; CST; rabbit mAb (C13F9); #2796; anti-Lyn; CST; mouse mAb (5G2); #4576, anti- Gapdh; Abcam [6C5]; #Ab8245


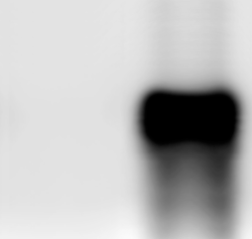

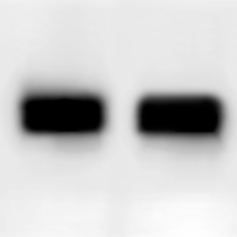

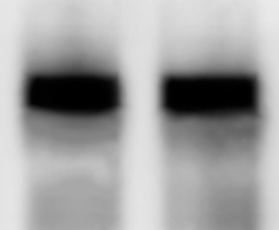

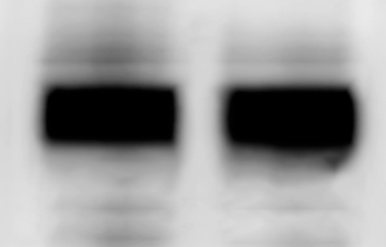
anti-Src anti-Fyn anti-Lyn anti-E

**
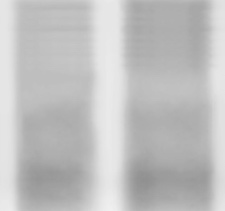
**
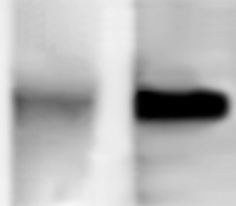
 **
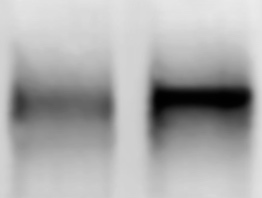

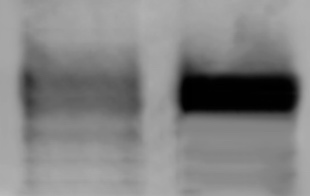
**

anti-Src anti-Fyn anti-Lyn anti-E

**
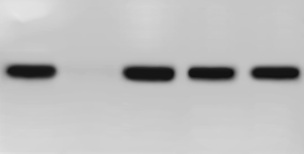

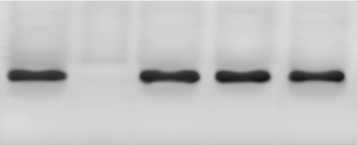

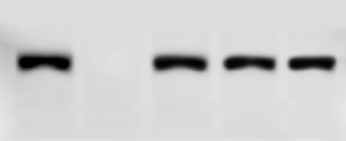

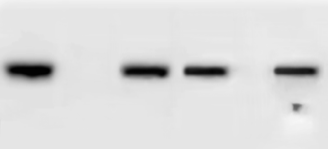
Figure 3a**: anti-Lyn; CST; mouse mAb (5G2); #4576, anti- Gapdh; Abcam [6C5]; #Ab8245

anti-Lyn (HepG2) anti-Lyn (HeLa) anti-Lyn (Huh7) anti-Lyn (Vero E6)

**
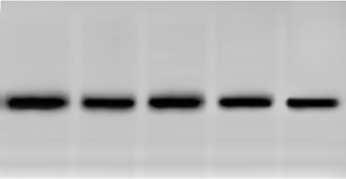

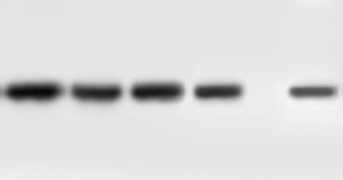

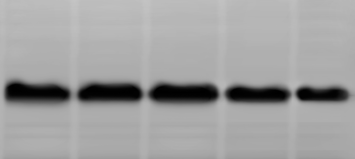

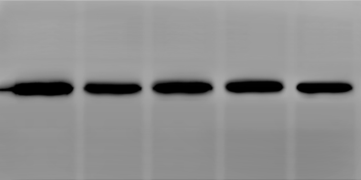
**

anti-Gapdh anti-Gapdh anti-Gapdh anti-Gapdh

**
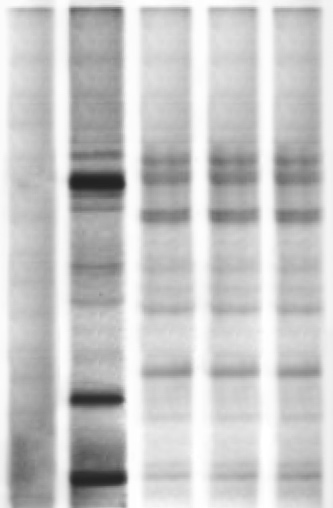

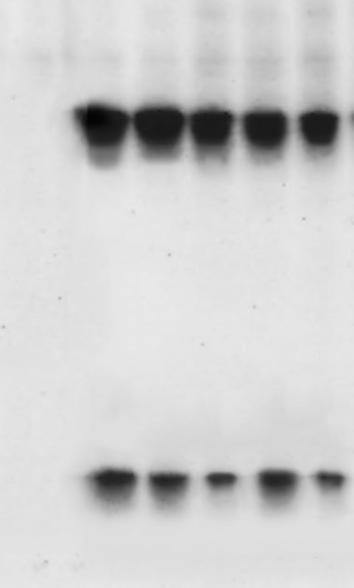
Figure 3g** **Figure 3h;** anti E mAb 4G2 prepared using hybridoma cells D1-4G2-4-15 (ATCC)

[^35^S] virus particles

**Figure 3j**: anti E mAb 4G2; anti-Gapdh Abcam [6C5]; #Ab8245


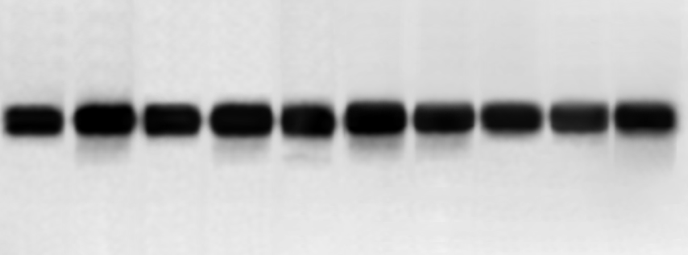

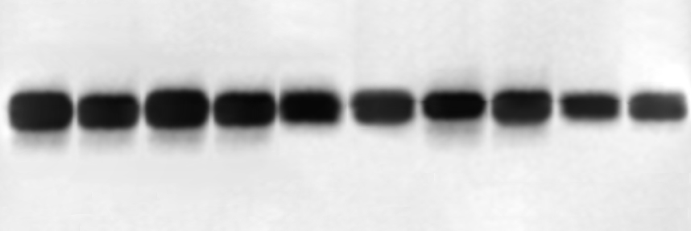
Anti-E (supernatants)

Anti-E (lysate)


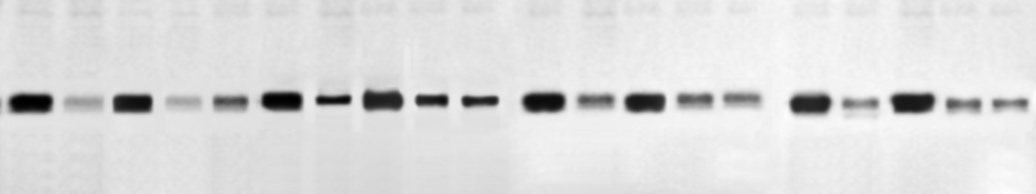
anti-Gapdh


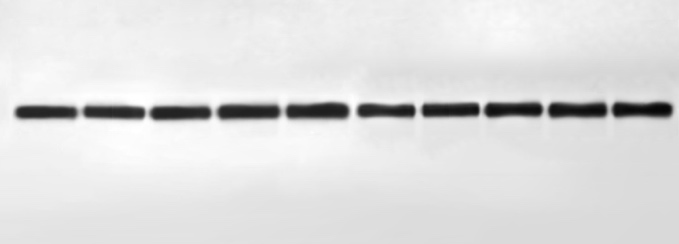

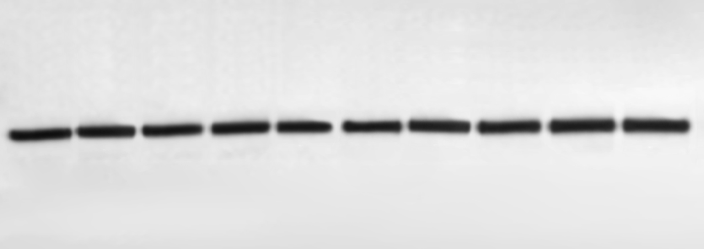
**
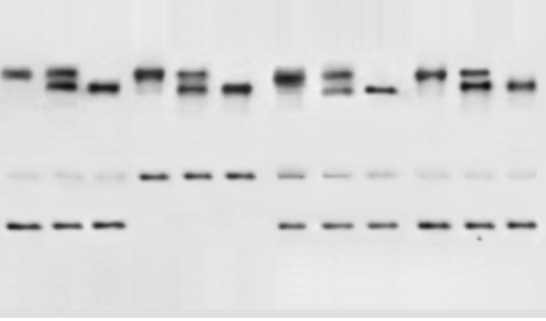

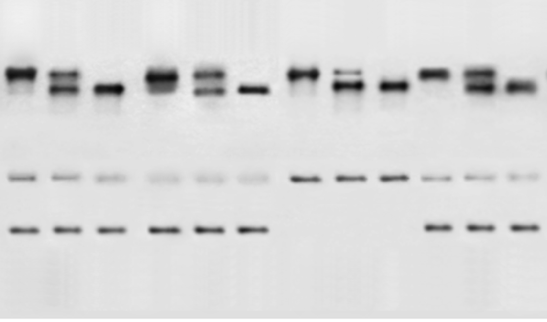

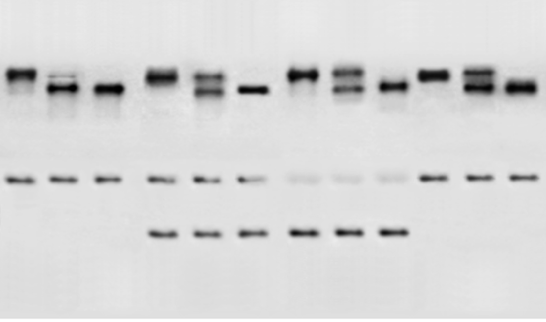
Figure 4b** anti E mAb 4G2 prepared using hybridoma cells D1-4G2-4-15 (ATCC)

anti E mAb

**Figure 4d:** anti E mAb

**
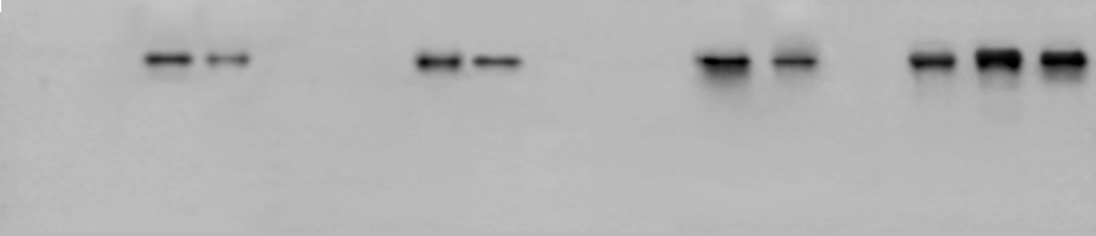
**

Anti-E (surface)


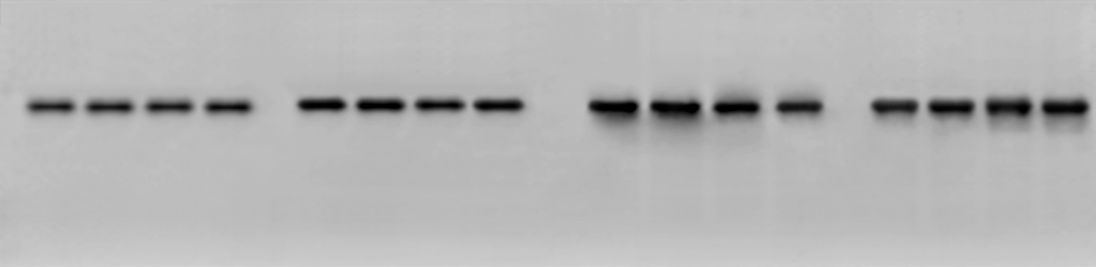


Anti-E (lysate)


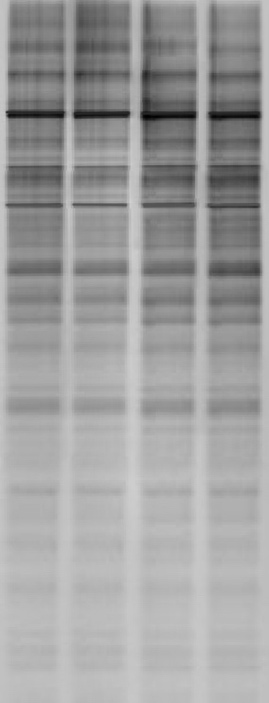
**Figure 4f**: Anti-PDI [RL90]; Abcam ab2792

**Figure 4g:** Anti-VSVG; Santa Cruz; sc-365019


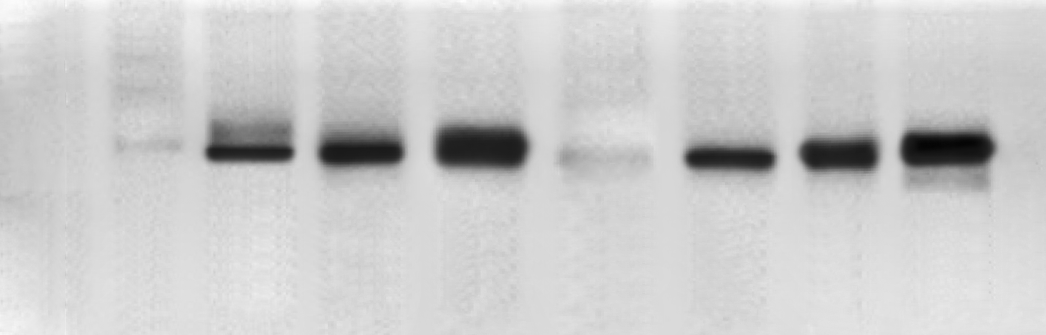


Anti-VSV-G

[35S]secreted proteins anti-VSV-G (intracellular)

**
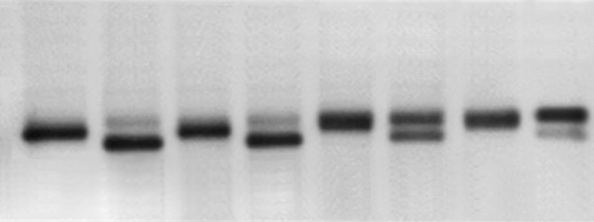
**

**
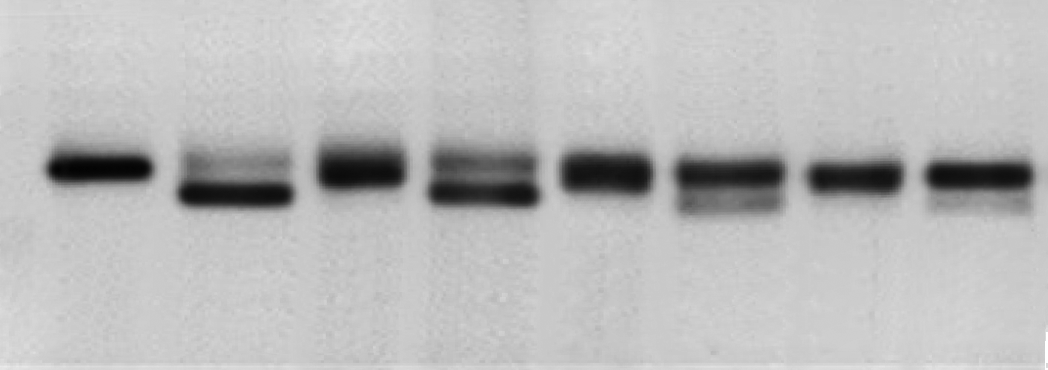
**

**Figure 4h**: Anti-influenza HA; mouse mAb [AT1B7]; # ab139361


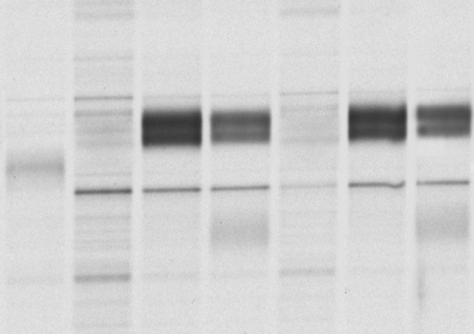

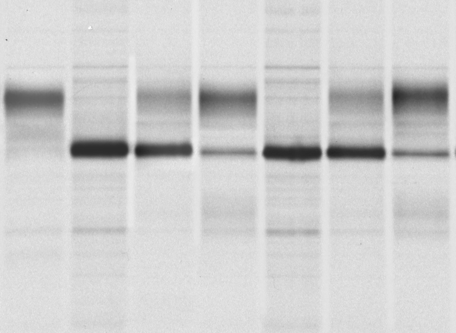


IP: Anti-influenza HA (surface) IP: Anti-influenza HA (intracellular);

[^35^S]cys/met labelled

**Figure 5**: Anti-Hrd (rabbit monoclonal, Abcam, ab170901), anti-sec22b (rabbit monoclonal, Abcam, ab181076), anti-ERGIC53 (rabbit monoclonal, Abcam, ab125006), anti-Tfr (rabbit polyclonal, Abcam, ab80194), anti-LC3 (rabbit monoclonal, Abcam, ab192890), anti-Lyn, anti-Src (CST; rabbit mAb; 36D10), anti-Fyn (CST; rabbit mAb; #4023) anti-Lyn (CST; rabbit mAb (C13F9); #2796; anti-Lyn; CST; mouse mAb (5G2); #4576), anti-Lamp1 (rabbit polyclonal, Abcam, ab24170), anti-Lamp2 (mouse monoclonal, Abcam, ab25631)

**Figure 5a**


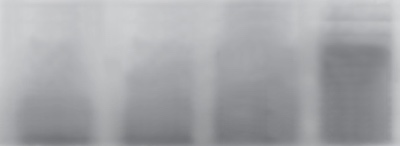

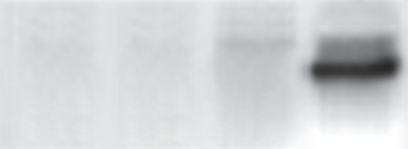

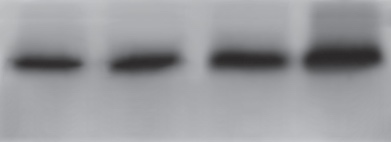


anti-Fyn IP anti-Src IP anti-Lyn IP


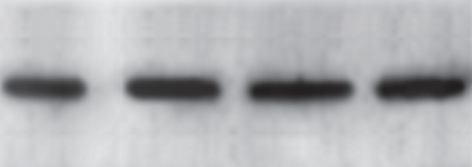


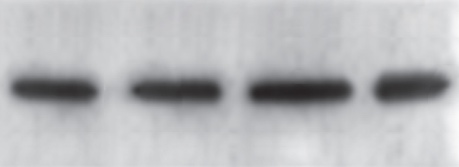

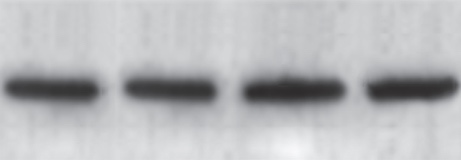
 anti-SFK


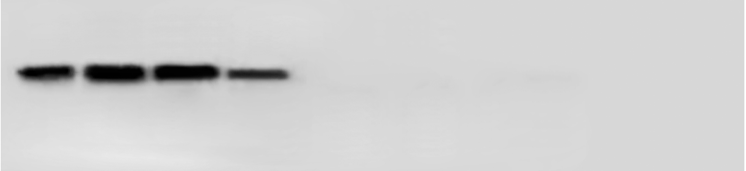
**Figure 5d**


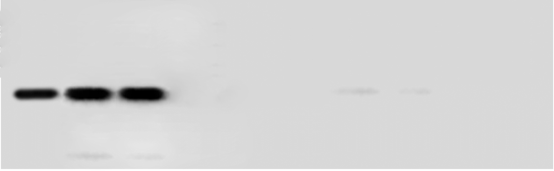
anti-Hrd1 anti-Sec22B


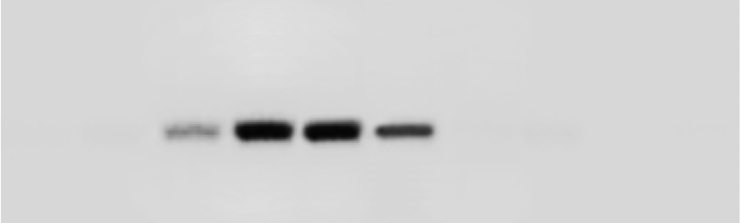


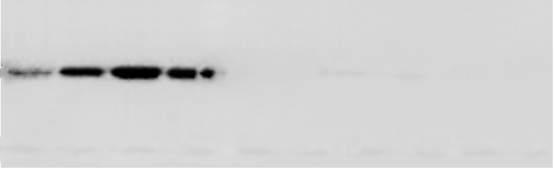


Anti-Ergic53 anti-Tfr


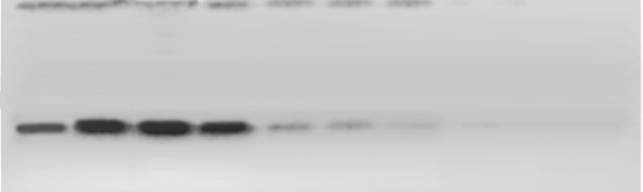

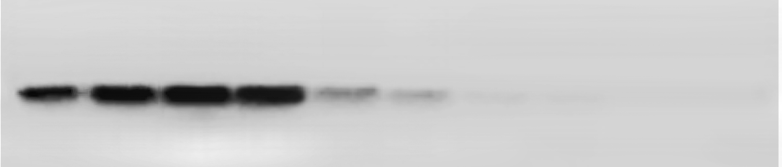
Figure 5c


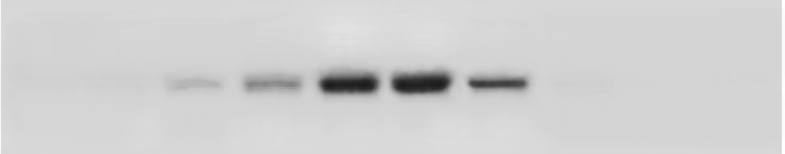

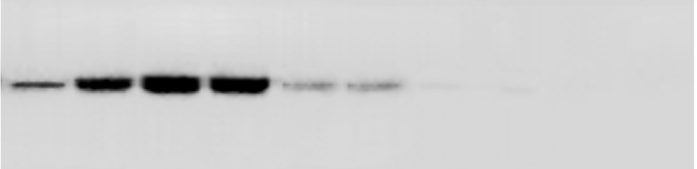
Anti-Hrd1 anti-sec22b


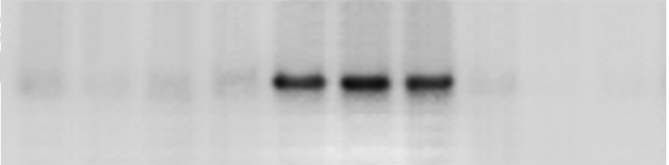

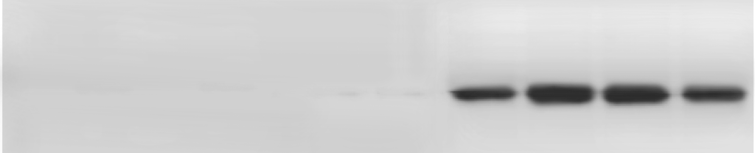
Anti-Ergic53 anti-Tfr


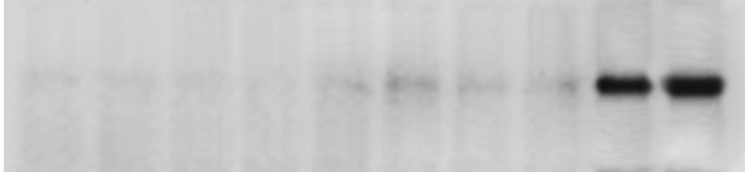
Anti-Rab11 anti-Golgin97


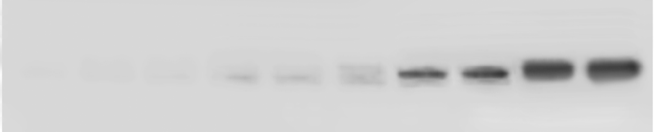


Anti-Lamp1 anti-Lamp2


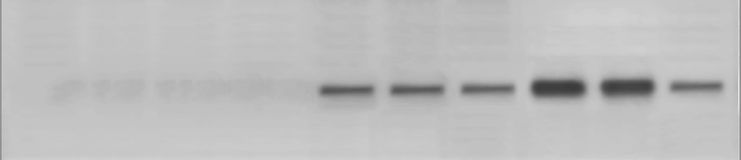

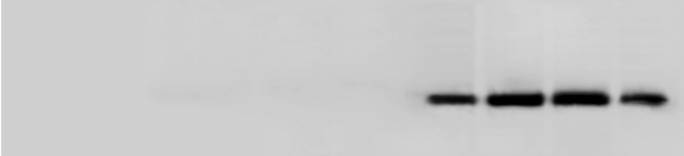


Anti-Lyn anti-Lyn


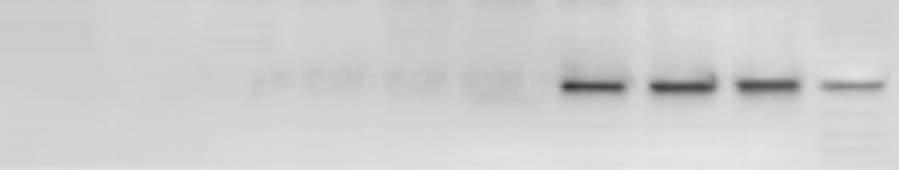


Anti-Src


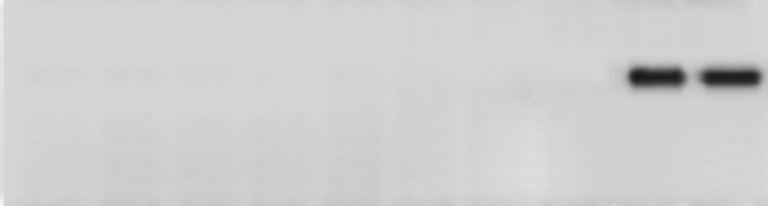


Anti-Lamp1


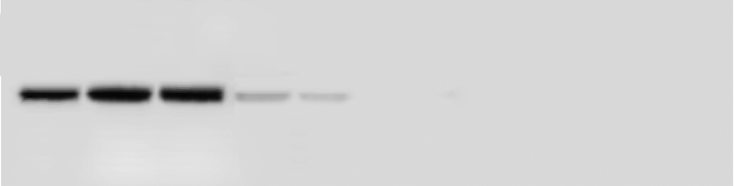

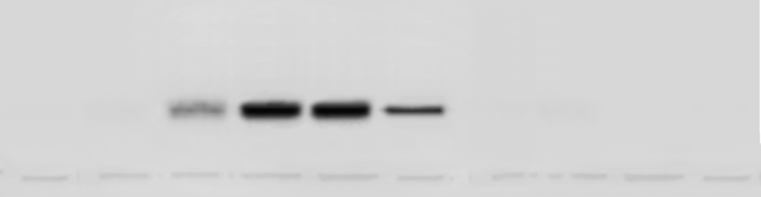
Figure 5e

Anti-Hrd1 (Lyn C3S) anti-Tfr (Lyn C3S)


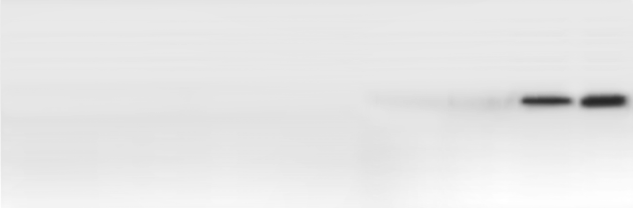

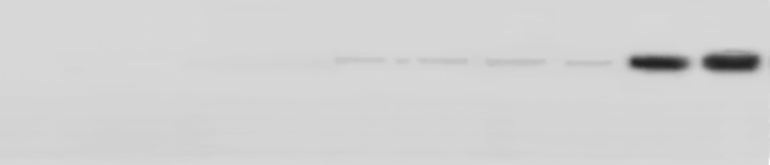


anti-E (Lyn C3S) anti-Lamp1 (Lyn C3S)


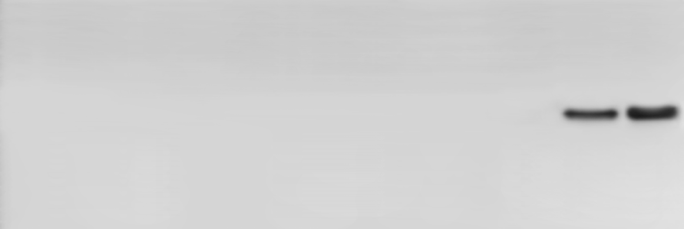


anti-Lyn (Lyn C3S)


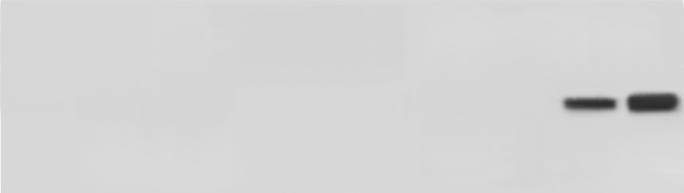

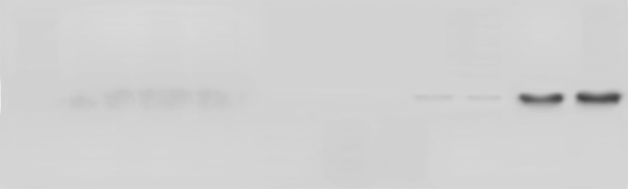

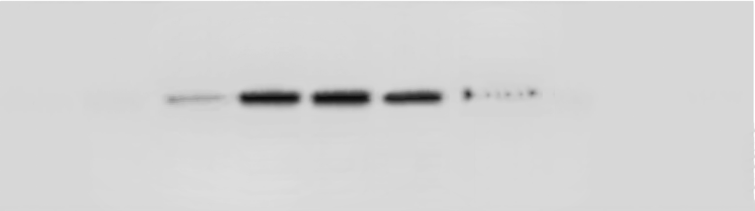

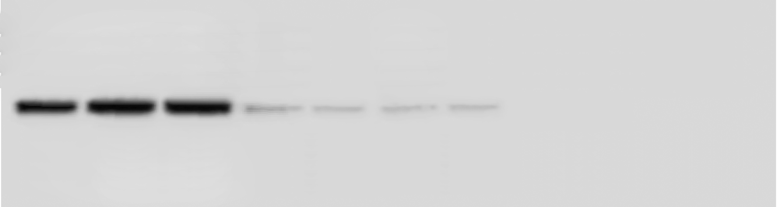
anti-Hrd1 (C468A) anti-Tfr (C468A)

anti-E (C468A) anti-Lamp1 (C468A)


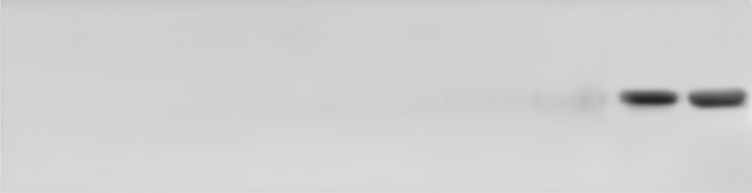


anti-Lyn (C468A)


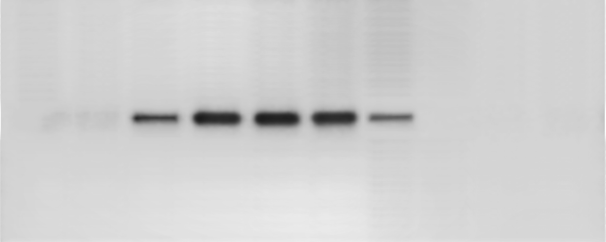


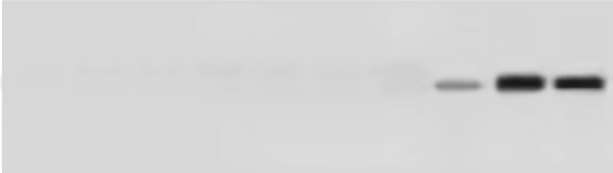


anti-E (Lyn wt) anti-E (Lyn^-/-^)

anti-Lamp1 anti-Lamp1

Figure 5f

Anti-E anti-Gapdh

Anti-E anti-Gapdh

**Figure 6b** anti E mAb 4G2 prepared using hybridoma cells D1-4G2-4-15 (ATCC)

Anti-E

[^35^S]cysteine/methionine labelled VLPs

Figure 6c

[^35^S]cysteine/methionine labelled VLPs

Anti-E

Figure 7h anti-Ulk1 (D8H5); CST#8054; anti-Fip200 (D10D11); Rabbit mAb; CST12436; anti-ATG13 (E1Y9V); CST#13468); anti-Rab8A (D22D8); CST#6975; anti-Rab27A Abcam# ab55667; anti-Rab11A (D4F5); CST#5589; anti-Rab7A (D95F2); CST#9367; anti-Grasp55 (E11); Sc365602; anti-Grasp65 (OTI5G8) Cat# MA5-25148; anti-Trim16; Abcam# ab251749; anti-Stx3; Abcam# ab4113; Anti-Stx4 (QQ17); Sc101301; anti-Stx17; thermofisher cat#17815-1-AP

Anti-Ulk1 Anti-Fip200

Anti-Atg13 Anti-Gapdh

Anti-Rab8A anti-Rab27A

anti-Rab11A anti-Rab7

Anti-Gapdh

Anti-Grasp55 Anti-Grasp65

Anti-Trim16 Anti-Gapdh

Anti-Stx3 Anti-Stx4

Anti-Stx17 Anti-Gapdh

Supplementary figures

Figure S1a

Anti-pSFK anti-E

Anti-SFK anti-Gapdh

Figure S2a

anti-pSFK anti-SFK

Anti-Gapdh

Figure S2g

Anti-E anti-E

Anti-Gapdh

Figure S3a

Anti-Src anti-Fyn

Anti-Lyn anti-Gapdh

Figure S3f

Anti-Src anti-Fyn

Anti-Lyn anti-Gapdh

Figure S4a Figure S4c

Anti-E anti-E

Anti-Gapdh

[^35^S]VLPs

Figure S5a

Anti-Ulk1 anti-Lyn anti-Gapdh

anti-Grasp55 anti-Grasp65 Trim16

anti-Stx3 anti-Stx4 anti-Stx17

Figure 7e

Anti-LC3 anti-Rab11 anti-PrM

Anti-E

Anti-LC3 anti-LC3

Figure S8a

anti-Lck

anti-Gapdh­­

Figure S8d

anti-Hrd1 anti-Tfr

anti-E anti-Lck (un-induced)

anti-Lamp1

anti-Hrd1 anti-Tfr

anti-E anti-Lck

Anti-Lamp1

Anti-Hrd1 anti-Tfr

anti-E anti-Lck

anti-Lamp1

anti-Hrd1 anti-Tfr

anti-E anti-Lck

Anti-Lamp1
